# Effects of Dwarf Mistletoe on Stand Structure of Lodgepole Pine Forests 21-28 Years Post-Mountain Pine Beetle Epidemic in Central Oregon

**DOI:** 10.1371/journal.pone.0107532

**Published:** 2014-09-15

**Authors:** Michelle C. Agne, David C. Shaw, Travis J. Woolley, Mónica E. Queijeiro-Bolaños

**Affiliations:** 1 Department of Forest Engineering, Resources, and Management, Oregon State University, Corvallis, Oregon, United States of America; 2 Departamento de Ecología y Recursos Naturales, Facultad de Ciencias, Universidad Nacional Autónoma de México, Mexico City, DF, Mexico; Swiss Federal Research Institute WSL, Switzerland

## Abstract

Lodgepole pine (*Pinus contorta*) forests are widely distributed throughout North America and are subject to mountain pine beetle (*Dendroctonus ponderosae*) epidemics, which have caused mortality over millions of hectares of mature trees in recent decades. Mountain pine beetle is known to influence stand structure, and has the ability to impact many forest processes. Dwarf mistletoe (*Arceuthobium americanum*) also influences stand structure and occurs frequently in post-mountain pine beetle epidemic lodgepole pine forests. Few studies have incorporated both disturbances simultaneously although they co-occur frequently on the landscape. The aim of this study is to investigate the stand structure of lodgepole pine forests 21–28 years after a mountain pine beetle epidemic with varying levels of dwarf mistletoe infection in the Deschutes National Forest in central Oregon. We compared stand density, stand basal area, canopy volume, proportion of the stand in dominant/codominant, intermediate, and suppressed cohorts, average height and average diameter of each cohort, across the range of dwarf mistletoe ratings to address differences in stand structure. We found strong evidence of a decrease in canopy volume, suppressed cohort height, and dominant/codominant cohort diameter with increasing stand-level dwarf mistletoe rating. There was strong evidence that as dwarf mistletoe rating increases, proportion of the stand in the dominant/codominant cohort decreases while proportion of the stand in the suppressed cohort increases. Structural differences associated with variable dwarf mistletoe severity create heterogeneity in this forest type and may have a significant influence on stand productivity and the resistance and resilience of these stands to future biotic and abiotic disturbances. Our findings show that it is imperative to incorporate dwarf mistletoe when studying stand productivity and ecosystem recovery processes in lodgepole pine forests because of its potential to influence stand structure.

## Introduction

Lodgepole pine (*Pinus contorta* Dougl. ex Loud.) forests are widely distributed throughout western North America [1] and are subject to widespread mortality by mountain pine beetle (*Dendroctonus ponderosae* Hopkins) throughout their range. Although this bark beetle is native to lodgepole pine forests, it has caused landscape-scale losses of mature lodgepole pine in British Columbia and the Intermountain West in the last two decades, prompting concern regarding ecosystem function following disturbance events of this magnitude [2]. Recent studies have shown that epidemic disturbance by mountain pine beetle influences many ecosystem functions, including carbon sequestration [3], hydrology and nutrient cycling [4], fire hazard [5], and stand regeneration [6]. Stand structure, which affects many of these processes, is also dramatically influenced by mountain pine beetle outbreaks, undergoing rapid changes through time after an epidemic [7], [8].

The effect of mountain pine beetle epidemics on stand structure is highly dependent upon the time since beetle (TSB), as post-epidemic stands go through several structural phases over time [8]. Mountain pine beetle epidemics typically remove the largest cohort of trees and leave the suppressed and intermediate cohorts, as well as trees with low vigor, thin phloem, and dwarf mistletoe [9], [10]. Stand density is greatly decreased immediately post-epidemic and declined by over 50% as compared with pre-epidemic stands during a recent epidemic in Colorado [11], [12]. However, by 20 to 30 years TSB, stand density has been shown to recover to pre-epidemic conditions in some areas [13], and may surpass the density of stands which have not been recently attacked [14]. Similarly, stand basal area is immediately reduced by up to 70% post-epidemic [11], [12], but reaches about 60% of pre-epidemic basal area by 25–30 years TSB [13] and is predicted to recover fully by 80 years TSB in Colorado lodgepole pine stands [15]. Tree size distribution is immediately skewed toward the small size classes post-epidemic with the removal of the large trees which are the preferred mountain pine beetle host in epidemics [9]. Overall stand height and average diameter at breast height (DBH) are also decreased following a mountain pine beetle epidemic [9], [12].

Forest stand structure of lodgepole pine influences hydrologic function [16], biodiversity [17], stand productivity [18], and ecosystem resistance and resilience to insect outbreaks [11], [19], fire [20], fungal infections [21], as well as complex interactions between biotic and abiotic disturbances [22]. Because stand structure drives many forest processes, interest in the influence of mountain pine beetle epidemics on stand structure has recently increased [6], [13]. However, less attention has been focused on the investigation of other factors which might influence the stand structure of post-mountain pine beetle epidemic environments. To fully understand the processes which occur in a mountain pine beetle-disturbed forest, other factors affecting stand structure must be considered. The presence of compound disturbances (*sensu* [23]) in ecosystems necessitates that multiple disturbance effects are accounted for simultaneously, as summing their individual effects may not represent their combined effects. Although complex interactions surrounding disturbance dynamics have begun to be quantified [24], [25], integration of multiple disturbances remains a key gap in ecosystem modeling [26], [27].

**Figure 1:**
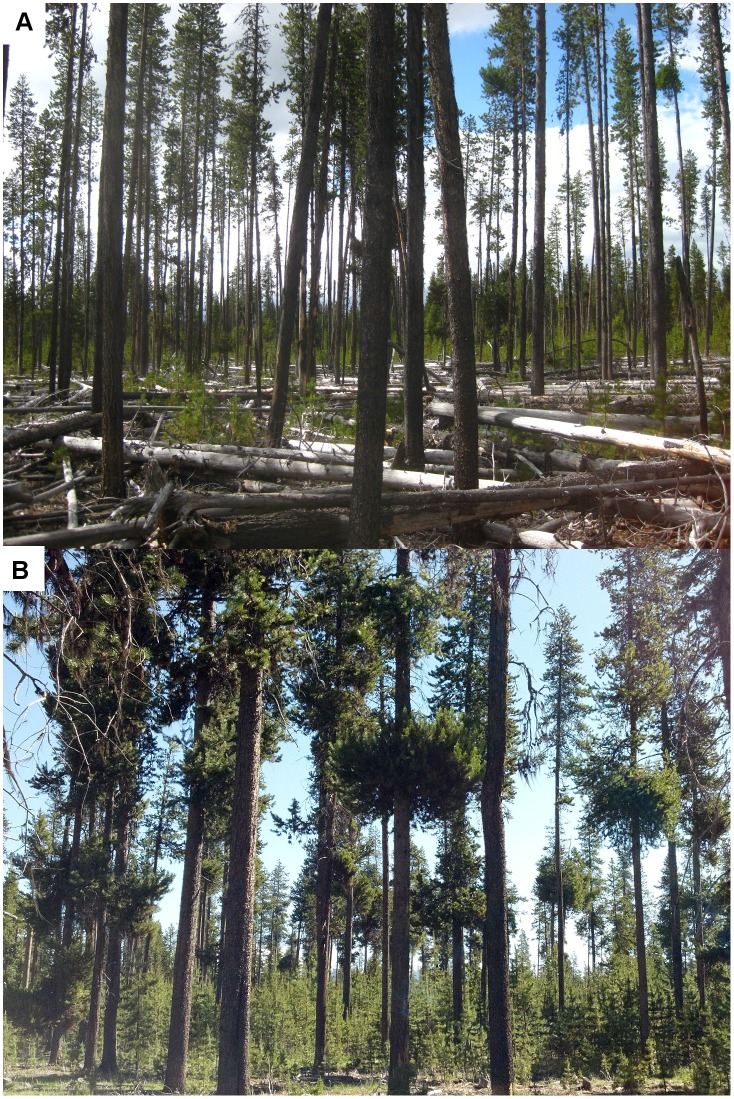
Forest structures of lodgepole pine stands 21–28 years after a mountain pine beetle epidemic. Pictured are stands A) without dwarf mistletoe and B) with severe dwarf mistletoe and high levels of witches’ brooming.

Dwarf mistletoes (*Arceuthobium* spp.) are a group of obligate hemiparasites which obtain the majority of their carbohydrates, nutrients, and water from their hosts [28], [29], leading to host growth loss, lowered vigor, and higher susceptibility to mortality when trees are severely infected [30]. Lodgepole pine dwarf mistletoe (*Arceuthobium americanum* Nutt. ex Engelm.), is a widespread pathogen of lodgepole pine, occurring throughout the range of its host [31]. In addition to growth loss, moderate to severe infection by *A. americanum* often induces host deformities by forming dense branch masses called witches brooms (Figure 1), which concentrate biomass in infected branches and act as nutrient sinks [32]. Although not all dwarf mistletoe species induce witches brooms as a symptom of infection, the individual structure of lodgepole pine crowns infected with *A. americanum* is significantly impacted via this mechanism, leading to shorter live crowns, which are skewed to the lower strata of the canopy [33]. Furthermore, decreases in average lodgepole pine diameter and height, particularly within larger size classes, have been observed with increased dwarf mistletoe infection levels [34], [35], [36], [37]. Lodgepole pine dwarf mistletoe also influences several aspects of overall stand structure in severely infected lodgepole pine forests, in addition to individual crown structure. Stand density has been shown to increase five-fold in severely infected stands [36]. The increase in stand density is attributed to a shift in tree size distribution to smaller size classes, with increased densities of suppressed trees and decreased densities of dominant trees in stands with high levels of dwarf mistletoe as compared with stands without dwarf mistletoe [33], [36], [38].

Although lodgepole pine dwarf mistletoe and mountain pine beetle are both known to individually influence stand structure, previous dwarf mistletoe research in lodgepole pine has not taken time since the previous mountain pine beetle epidemic into account [33], [36], [38], [39]. Similarly, previous studies on mountain pine beetle have not accounted for dwarf mistletoe [6], [11], [13], [15]. However, a recent study demonstrated that the interaction between southwestern dwarf mistletoe (*Arceuthobium vaginatum* subsp. *cryptopodum* Engelm.) and mountain pine beetle influences stand structure in ponderosa pine forests in Colorado [40]. This indicates that the interaction between dwarf mistletoe and bark beetles may influence the ecology of other forest types and should be investigated.

Lodgepole pine dwarf mistletoe is frequently found in lodgepole pine stands which have recently experienced a mountain pine beetle epidemic. A random sample of 212 lodgepole pine dominated plots in central Oregon 2–31 years post-mountain pine beetle epidemic indicated that 72% of post-epidemic lodgepole pine stands in the area had some level of dwarf mistletoe infection (unpublished data). Light to moderate dwarf mistletoe infection was found in 53% of the plots while 19% of the plots had severe dwarf mistletoe infection. Although there is evidence that dwarf mistletoe influences stand structure, few studies have demonstrated the effect of dwarf mistletoe on stand structure using randomized sites, so inference is typically limited to sites with severe dwarf mistletoe. The large percentage of post-mountain pine beetle epidemic plots with light to moderate dwarf mistletoe infection indicate that understanding the effects of lower infection levels, in addition to the effects of high severity infections, is important to understanding its influences on stand dynamics. Mountain pine beetle and lodgepole pine dwarf mistletoe co-occur with high frequency, so the response of stand structure to both mountain pine beetle and dwarf mistletoe must be considered simultaneously to understand structural effects occurring on the landscape.

We chose to specifically investigate the interaction between mountain pine beetle and lodgepole pine dwarf mistletoe in stands 21–28 years TSB. We chose this time period because central Oregon experienced a mountain pine beetle epidemic in the 1980’s spatially analogous to that which is currently occurring in British Columbia and the Intermountain West. Therefore, the results from this study could provide valuable insight to future stand structure in other areas post-mountain pine beetle epidemic. Differences exist throughout the range of this forest type, as in central Oregon the lodgepole pine stands often exist as climax, un-even aged stands [41], while in most other regions lodgepole pine is an early successional species that grows primarily in even-aged cohorts [1]. However, mountain pine beetle rarely causes 100% mortality of mature lodgepole pine at the stand level [42] and advance lodgepole pine regeneration is often a large component of the understory, even in seral stands [43]. Uneven stand structure and significant amounts of lodgepole pine regeneration have been noted in British Columbia [44], [45], Colorado [11], [14], Wyoming [6], Idaho, Utah, and Montana [43] lodgepole pine stands after a mountain pine beetle epidemic. The results from this study may inform future conditions in uneven-aged lodgepole pine stands in these areas that have recently experienced widespread mortality.

To address the interaction between dwarf mistletoe and mountain pine beetle we asked: “How does stand structure of lodgepole pine forests 21–28 years post-mountain pine beetle epidemic change with varying levels of dwarf mistletoe infection?” We identified several important metrics for assessment of stand structure: stand density, stand basal area, canopy volume, proportion of lodgepole pine in dominant/codominant, intermediate, and suppressed cohorts, and average height and DBH of trees within cohorts. Each of these characteristics is affected by either dwarf mistletoe, mountain pine beetle, or both of these disturbance agents. We hypothesized that in lodgepole pine stands 21–28 years TSB: 1) stand density increases with increasing dwarf mistletoe severity, 2) stand basal area and canopy volume decrease as dwarf mistletoe severity increases, 3) as dwarf mistletoe severity increases, larger proportions of lodgepole pine are represented by cohorts of suppressed trees and smaller proportions of lodgepole pine are represented by cohorts of dominant and codominant trees, and 4) average height and diameter of all cohorts decreases as dwarf mistletoe severity increases.

## Materials and Methods

### Study Area

**Figure 2:**
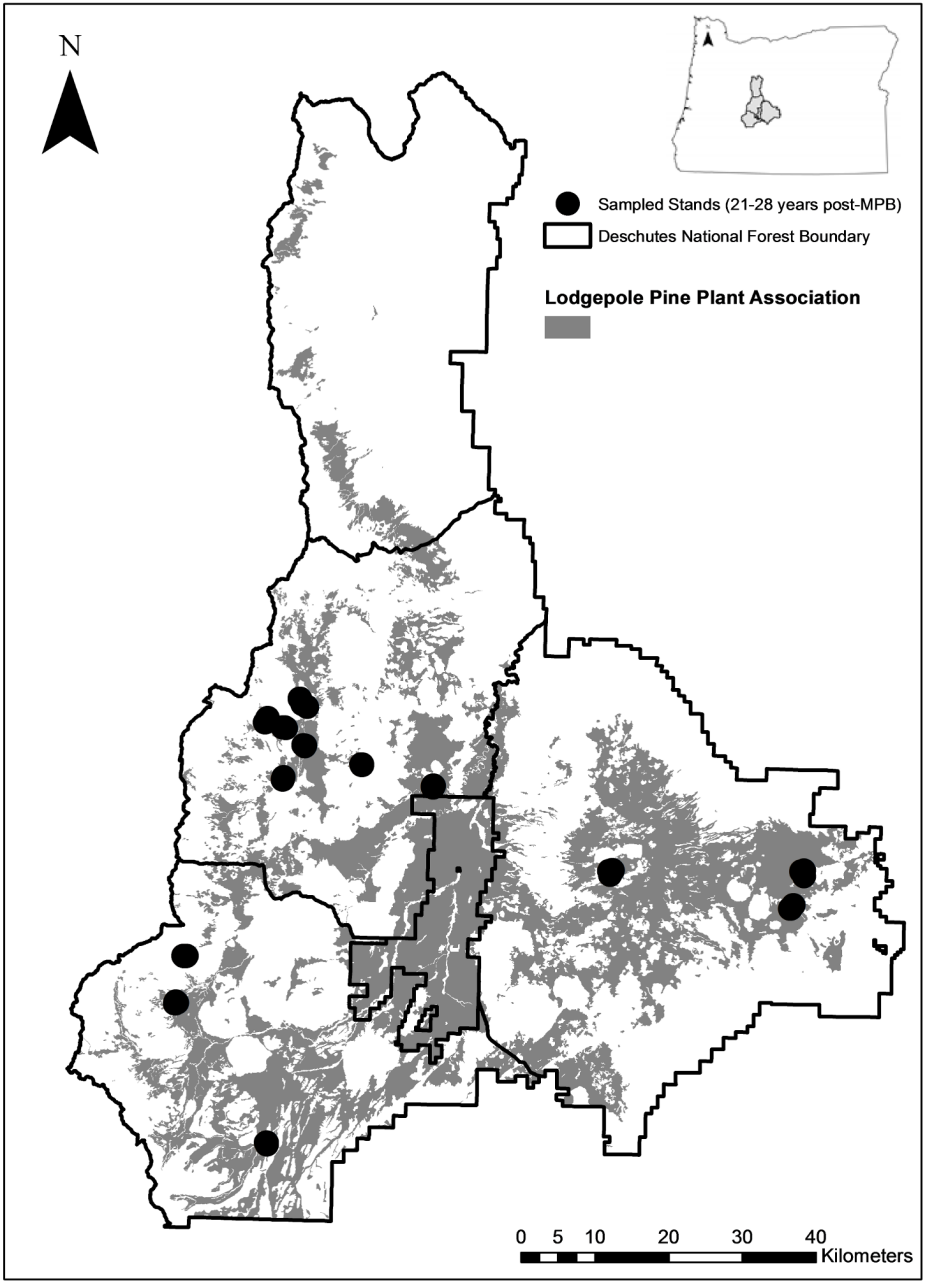
Study area map. Deschutes National Forest, Oregon boundary with sampled stands and lodgepole pine plant associations.

The study area for this research is located in central Oregon within the Deschutes National Forest. The Deschutes National Forest is located on the east side of the Cascade Mountains, covering an area of approximately 728,000 hectares (Figure 2). Stands were chosen within the edaphic and topoedaphic climax lodgepole pine zones according to the plant association guide for the area [41], [46]. In this area, the ecological site characteristics of the climax lodgepole pine type are relatively uniform, characterized by pumice soils and flat to gently rolling topography which often results in cold air drainage [47]. The lodgepole pine zone is located between 1,200 and 1,525 meters elevation with mean annual temperatures ranging from 4.3 to 5.8°C and mean annual precipitation ranging from 38 to 89 cm depending upon the specific plant association [41]. The Wickiup Dam climate station (the most representative climate station for the study area) showed average daily temperatures ranging from −2.2°C in January to 18.3°C in July [48].

Measured stands were restricted to areas 21–28 years post mountain pine beetle epidemic to control for the effect of mountain pine beetle on stand structure. The year of initiation of each mountain pine beetle mortality event was determined using Aerial Detection Survey (ADS) data [49]. Areas with known past management or recent fire were excluded from sampling. Stands were characterized by large amounts of coarse wood, few standing snags, and dense lodgepole pine regeneration. The overstory was typically comprised of lodgepole pine too small to support a mountain pine beetle brood at the time of the previous epidemic that subsequently released after the mortality of the majority of the previous overstory.

A minimum of 70% of live trees at each plot measured were lodgepole pine. Other tree species found at the sites varied with elevation. Ponderosa pine (*Pinus ponderosa* Dougl. ex Laws.) was present at lower elevations, while white fir (*Abies concolor* (Gord. and Glend.) Lindl. ex Hildebr.), grand fir (*Abies grandis* (Dougl. ex D. Don.), mountain hemlock (*Tsuga mertensiana* (Bong.) Carrière), and whitebark pine (*Pinus albicaulis* Engelm.) were located at higher elevations. Engelmann spruce (*Picea engelmanni* Parry ex Engelm.) and western white pine (*Pinus monticola* Dougl. ex D. Don) were occasionally present within the study area as well.

### Stand Selection

Stands were selected based on a network of 119 plots established in 2010 and 2011 within post-mountain pine beetle epidemic climax lodgepole pine, 2 to 31 years TSB, in the Deschutes National Forest (unpublished data). The network of plots was designed using a spatially balanced random sampling design [50] with the purpose of broadly characterizing change in stand structure and fuels across lodgepole pine forests after a mountain pine beetle mortality event in central Oregon. However, individual stands were not intensively sampled. Therefore, stand polygons were drawn in ArcGIS 9.3 [51] around the 38 original plots which were 21 to 28 years TSB to more intensively sample the areas in which the original plots were located. Because some stands contained more than one of the original plots, or were not large enough to accommodate our sampling design, a total of 26 stands were available for sampling. Stand boundaries were drawn based on the presence of a climax lodgepole pine plant association, ADS data regarding the most recent mountain pine beetle epidemic, and GIS layers from the Deschutes National Forest regarding past management activities, to ensure that the stand polygons were ecologically consistent with the original plot.

Within each polygon, three GPS points were selected as beginning points for the plots using a spatially balanced random sampling design [50]. A random azimuth used for orientation of the plot was generated for each point. Each point was checked in the field to ensure that the associated plot was dominated by lodgepole pine, had past influence of mountain pine beetle, and had no sign of past management or recent fire. If any of these criteria were not met for a given point, a randomly selected replacement GPS point within the site boundary was used in its place.

### Plot Layout and Sampling Protocol

During the summer of 2012, a total of 13 stands were randomly selected from the 26 stands available for sampling. We established three 75 m×10 m (0.075 ha) belt transects randomly located and oriented within each of the 13 stands, for a total of 39 plots (Table S1). This layout was chosen in order to obtain spatially explicit canopy data [52]. Upon establishment of each plot, UTM coordinates were taken at each end of the belt transect using a Trimble unit. From these points, slope and aspect were recorded. Species, vigor rating, crown class (dominant, codominant, intermediate, or suppressed), an X, Y coordinate, and DBH were determined for all live trees (DBH >/ = 5.0 cm) within the plots. In addition, tree height (to highest live crown), height to crown base (defined as the lowest live foliage), and crown width were measured to the nearest 0.1 meters.

Each live tree (DBH≥5 cm) was given a dwarf mistletoe severity rating using the Hawksworth Six-Class Dwarf Mistletoe Rating (DMR) System [53]. This system is based upon a rating of the number of branches visibly infected by dwarf mistletoe within each third of the tree’s live crown. Scores range from 0 (no visible infections) to 6 (50% or more of the branches in each third of the tree have visible infections). Brooms influence DMR estimations [54], so we observed crowns with binoculars and based DMR ratings on the presence of dwarf mistletoe plants themselves, rather than associated symptoms such as witches’ brooms. Tree crowns are open and clearly visible in this forest type, providing a high level of confidence in the accuracy of our DMR estimations. DMR of all lodgepole pine were averaged over the plot to obtain a plot-level DMR. Hereafter, DMR refers to dwarf mistletoe severity rating at the plot-level, rather than the individual tree-level.

### Ethics Statement

No permits were required to complete the field sampling for this study, as all sites were located on public Forest Service land, managers were informed of the research, and no destructive sampling was conducted. No protected species were sampled for this study.

### Stand Structure Metric Calculations

Stand density is defined as the total number of stems DBH≥5.0 cm/ha, calculated at the plot-level (Table 1). Stand basal area is defined as the sum of live tree basal area of lodgepole pine DBH>5.0 cm (m^2^/ha) in each plot using the formula: 3.142*(DBH/200)^2^. Crown volume (m^3^) measurements were calculated for live trees (DBH≥5.0 cm) using measurements of crown length, width, and height taken in the field. A crown form factor (CFF) was then applied to each volume to simulate the shape of a lodgepole pine crown [55]. The idealized crown shape “fat cone” (CFF: 0.2945) was used for dense stands (>1000 stems/ha), while the idealized crown shape “paraboloid” (CFF: 0.3927) was used for moderate and open stands (<1000 stems/ha). Individual crown volumes were summed over each plot to obtain total canopy volume (m^3^).

**Table 1:** Characteristics of 39 lodgepole pine plots within 13 stands in the Deschutes National Forest, Oregon.

Stand	Plot	DMR	ProductivityClass	MPBMortality Class	Stand Density(stems/ha)	Stand BasalArea (m^2^)	CanopyVolume (m^3^)
CRL	1	2.78	L	L	1213	11.94	409.9
	2	2.93	L	L	680	6.57	266.4
	3	2.40	L	L	600	15.02	539.1
CRP	1	0	H	H	1120	21.29	1129.3
	2	1.02	H	H	880	16.55	1235.3
	3	0	H	H	1107	23.87	608.0
CRP2	1	1.81	H	L	1107	14.58	337.0
	2	2.45	H	L	893	14.99	313.9
	3	2.94	H	L	1187	15.26	265.9
CUL2	1	2.42	H	L	973	14.67	329.4
	2	2.35	H	M	1040	11.08	252.3
	3	2.38	H	H	827	7.70	316.7
CUL6	1	3.98	M	H	987	12.78	696.9
	2	3.70	M	L	1000	10.67	431.7
	3	2.59	M	L	1760	22.12	931.2
DES	1	0	M	M	600	19.91	1347.1
	2	0	M	M	720	16.18	1216.4
	3	0	M	M	493	15.00	1422.8
EFR	1	0	L	M	600	18.94	764.4
	2	0	L	M	387	19.25	1185.0
	3	0	L	M	893	23.04	640.0
EFR3	1	0	L	H	613	16.00	917.3
	2	0	L	L	373	14.09	905.8
	3	0	L	M	2053	34.09	734.2
LDES	1	0.54	M	M	680	9.64	332.6
	2	0	M	M	1053	21.78	1305.7
	3	0.73	M	M	1013	18.29	813.1
LVLK	1	1.11	H	H	880	7.70	282.4
	2	2.09	H	H	1227	15.08	934.9
	3	0	H	H	1093	7.84	341.3
ODL	1	2.50	L	L	827	16.53	390.5
	2	1.85	L	L	987	12.27	396.4
	3	2.88	L	L	800	11.70	387.7
PAU	1	2.36	H	M	1733	21.87	602.3
	2	2.38	H	H	2493	26.03	609.8
	3	1.80	H	H	1560	23.48	664.9
SNC	1	1.40	H	H	533	8.02	277.4
	2	1.26	H	H	413	10.51	376.7
	3	0.16	H	H	493	12.99	590.6

Note: Data are calculated from measures of trees DBH >/ = 5.0 cm. DMR = stand-level dwarf mistletoe rating; MPB = mountain pine beetle. Productivity class was determined by previously developed plant associations for the area. MPB Mortality class was determined using Aerial Detection Survey cumulative mortality data where L = 5–15 trees/acre, M = 15–25 trees/acre, H = 25–36 trees/acre killed by mountain pine beetle over the time period of the epidemic.

Cohorts were defined by tree crown class assigned in the field. Three cohorts were identified: dominant/codominant, intermediate, and suppressed. Very few trees in each plot were classified as “dominant,” and these trees were often not much taller than the trees that were classified as “codominant.” This lack of distinction suggested that it was not appropriate to refer to dominant and codominant as separate cohorts. Hereafter, they will be grouped as a single cohort. For each cohort, we calculated the proportion of total lodgepole pine (DBH≥5.0 cm) represented by that cohort (proportion in cohort), the average height of the cohort (cohort height), and the average DBH of the cohort (cohort diameter) at the plot-scale (Table S2). We included only lodgepole pine in our cohorts because we were interested in the response of each cohort to dwarf mistletoe rating. Although ponderosa pine, whitebark pine, and Engelmann spruce are known occasional hosts of *A. americanum* [30], we never observed infection of occasional hosts in our plots and consider them non-hosts for the purposes of this study.

### Model Selection and Data Analysis

To describe the responses of stand structure parameters to DMR, we used linear mixed models (LMMs) and generalized linear mixed models (GLMMs) in which the response and predictor variables were continuous. Plots were nested within stands to account for potentially high levels of within-stand structural variability. Several covariates were identified as potentially influential in the responses of various aspects of stand structure to DMR. Stand density, site productivity, and mountain pine beetle mortality were all determined to be potentially influential to stand structure, and could have the ability to mask an effect of DMR if not accounted for in the model. Because previous findings have indicated there is a relationship between stand density and DMR [36], stand density could only be used as a covariate if there was no significant relationship between stand density and DMR in our data. We found no evidence of this relationship; therefore, stand density was accounted for as a continuous covariate in our models. Plots were assigned to a site productivity category (low, moderate, or high) using plant association data [46] (Table 1). Plots were also assigned to a mountain pine beetle mortality category (low, moderate, or high) based upon total mountain pine beetle mortality density mapped from ADS from 1979 to 2008 (Table 1). These covariates were assessed for multicollinearity with DMR prior to model fitting.

To ensure that our final fitted models adequately captured ecological relationships of interest while retaining maximum parsimony, we used Bayesian Information Criterion (BIC) to select the most appropriate model(s) from a set of candidate models (Tables S3–S14). Models were fitted using the maximum likelihood method for each response [56]. A candidate model with the lowest BIC value (ΔBIC of 0) was considered to be the most appropriate model, however models with ΔBIC values of less than two were considered to perform equally well. In situations with more than one preferred model (ΔBIC<2), we chose to interpret the preferred model which included DMR as a predictor variable as the primary interest of this investigation is in the impact of dwarf mistletoe on stand structure. BIC weights and evidence ratios were also calculated for each set of candidate models to further assess the weight of evidence for each model in the set [56] (Tables S3–S14).

Assumptions of linearity, homoscedasticity and normality were assessed for all candidate models using standard diagnostics prior to model selection [57]. We logarithmically transformed the response variables canopy volume, cohort diameter of dominant/codominant, cohort diameter of intermediates, and cohort diameter of suppressed to correct for heteroscedasticity. We used LMMs to model the relationship between DMR and stand structure variables when these assumptions were met and refitted the preferred candidate models using the residual maximum likelihood method for final inference. The normality assumption is not met for proportion data, so we used binomial GLMMs to model the response of the proportion in cohort data. We assessed these models for overdispersion prior to model selection. We corrected for overdispersion, when necessary, by adding an individual-level random effect to the model [58]. We performed likelihood ratio tests to determine that the coefficients for the explanatory variables were different from zero.

Models with p-values below an α-level of 0.05 were interpreted to have strong evidence of a relationship. Models with p<0.10 were interpreted to have suggestive but inconclusive evidence of a relationship to lower the probability of making a Type II error given our sample size and the inherent variability of this study area. We report 95% confidence intervals for means estimated for LMMs and profile 95% confidence intervals for odds ratios estimated for GLMMs. We calculated marginal and conditional R^2^ values to describe goodness of fit for linear mixed models and generalized linear mixed models [59]. The marginal R^2^ represents variance explained by fixed factors alone and conditional R^2^ represents the variance explained by fixed and random factors. Both measures were calculated to understand the fit of these models. All analyses were performed using the program R, version 2.12.0 [60].

## Results

### Stand Attributes

**Figure 3:**
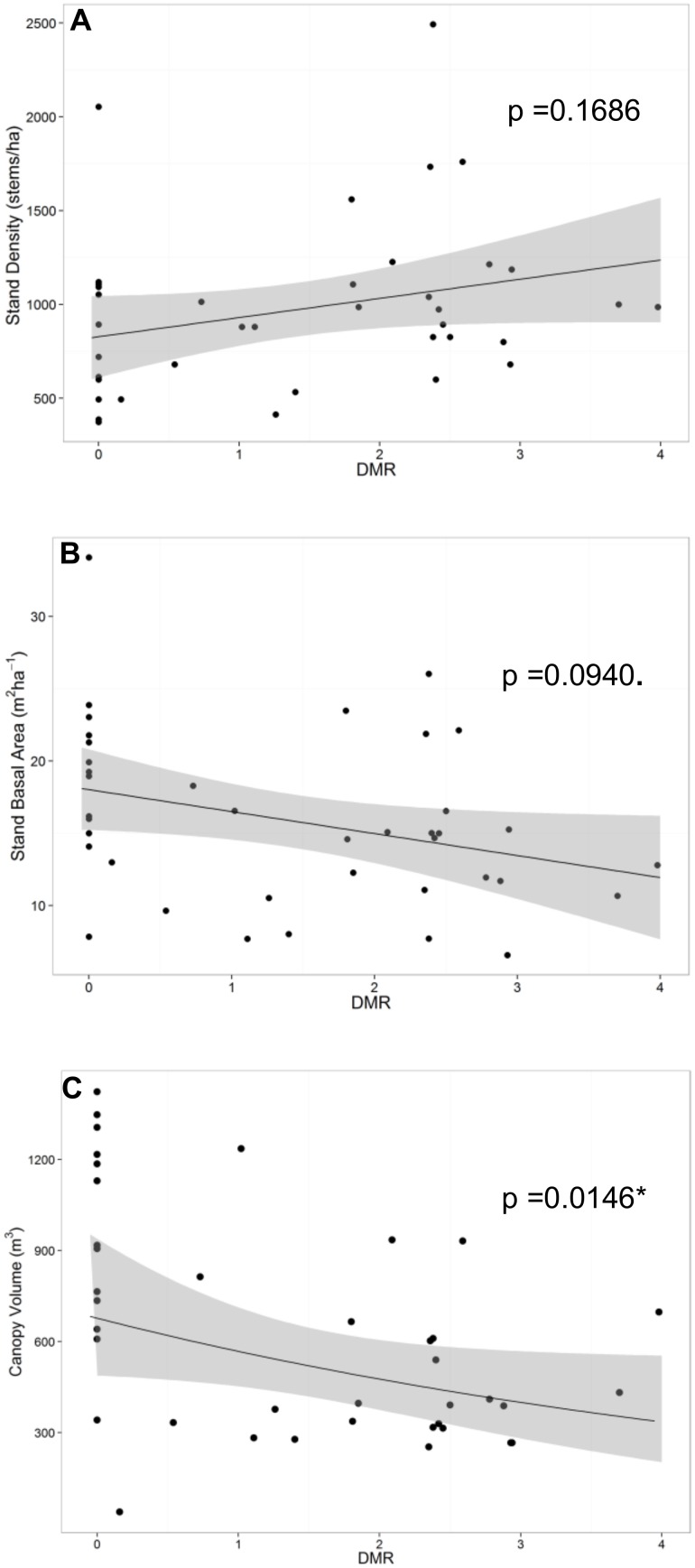
Linear mixed models of stand attributes on dwarf mistletoe rating. Scatterplots of linear mixed models of A) stand density (stems/ha), B) stand basal area (m^2^/ha), and C) backtransformed natural logarithm of canopy volume (m^3^) on dwarf mistletoe rating (DMR) with 95% confidence intervals.

For the each of the responses of stand density, stand basal area, and canopy volume, BIC showed that a model including the single continuous predictor variable of DMR was preferred (Tables S3, S4, S5). Although this model was selected for stand density, there was no evidence of a difference in stand density over the range of DMR (F_1,25_ = 2.01, p = 0.1686) (Table 2, Figure 3). This finding allowed us to consider stand density as a covariate in subsequent analyses. There was suggestive but inconclusive evidence that stand basal area decreased with increasing DMR (F_1,25_ = 3.04, p = 0.094) (Table 2, Figure 3). There was strong evidence that the natural logarithm of canopy volume decreased with increasing DMR in these stands (F_1,25_ = 6.890, p = 0.0146) (Table 2). There was an estimated 17.8% (95% CI: 4.1%, 29.4%) decrease in the median canopy volume for each unit increase in DMR (Figure 3).

**Table 2:** BIC preferred linear mixed models of stand characteristics.

Response variable	Model with parameterestimates (*SE*)	BIC	Marginal r^2^	Conditional r^2^	p value	
Stand density	Log(SD) = 6.652 (*0.128*) +0.094 * DMR (*0.066*)	−31.95	0.07	0.39	0.1686	
Stand basal area	SBA = 1.355 (*0.130*) –0.117 * DMR (*0.067*)	53.52	0.1	0.41	0.094	.
Canopy volume	Log(CV) = 6.615 (*0.146*) –0.195 * DMR (*0.074*)	59.11	0.21	0.53	0.0146	*
Proportion dominant	logit(PD) = −0.448 (*0.167*) −0.242 * DMR (*0.089*)	283.08	0.03	0.10	0.0153	*
Proportion intermediate	logit(PI) = −0.899 (*0.146*) –0.018 * SD (*0.011*)	238.02	0.01	0.02	0.1097	
Proportion suppressed	logit(PS) = −1.159 (*0.168*) +0.318 * DMR (*0.087*)	290.06	0.04	0.14	0.0022	**
Cohort height of dominants	CHD = 20.19 (*0.881*) –0.476 * DMR (*0.317*) −0.002 * SD (*0.001*)	175.34	0.31	0.56	0.0501	.
Cohort height of intermediates	CHI = 11.154 (*0.620*) –0.663 * DMR (*0.322*)	179.23	0.13	0.37	0.0501	.
Cohort height of suppressed	CHS = 5.676 (*0.295*) –0.450 * DMR (*0.150*)	113.39	0.26	0.57	0.0061	**
Cohort diameter of dominants	Log(CDD) = 3.37 (*0.055*) –0.0428 * DMR (*0.020*) −0.0002 * SD (*0.00004*)	−39.51	0.42	0.59	0.0074	**
Cohort diameter of intermediates	Log(CDI) = 2.76 (*0.050*) −0.0002 * SD (*0.00004*)	−44.62	0.3	0.56	0.0005	**
Cohort diameter of suppressed	Log(CDS) = 2.06 (*0.048*) −0.015 * DMR (*0.024*)	−33.95	0.02	0.47	0.5235	

Note: Log(SD) = mean of the natural logarithm of stand density; SBA = mean of stand basal area; Log(CV) = mean of the natural logarithm of canopy volume; logit(PD) = log odds that a lodgepole pine is in the dominant/codominant cohort; logit(PI) = log odds that a lodgepole pine is in the intermediate cohort; logit(PS) = log odds that a lodgepole pine is in the suppressed cohort; CHD = mean of the cohort height of dominants; CHI = mean of the cohort height of intermediates; CHS = mean of the cohort height of suppressed; CDD = mean of the natural logarithm of cohort diameter of dominants; CDI = mean of the natural logarithm of cohort diameter of intermediates; CDS = mean of the natural logarithm of cohort diameter of suppressed; DMR = stand level dwarf mistletoe rating; SD = stand density; BIC = Bayesian Information Criterion; ** = p<0.01; * = p<0.05; . = p<0.1.

### Cohort Attributes

**Figure 4:**
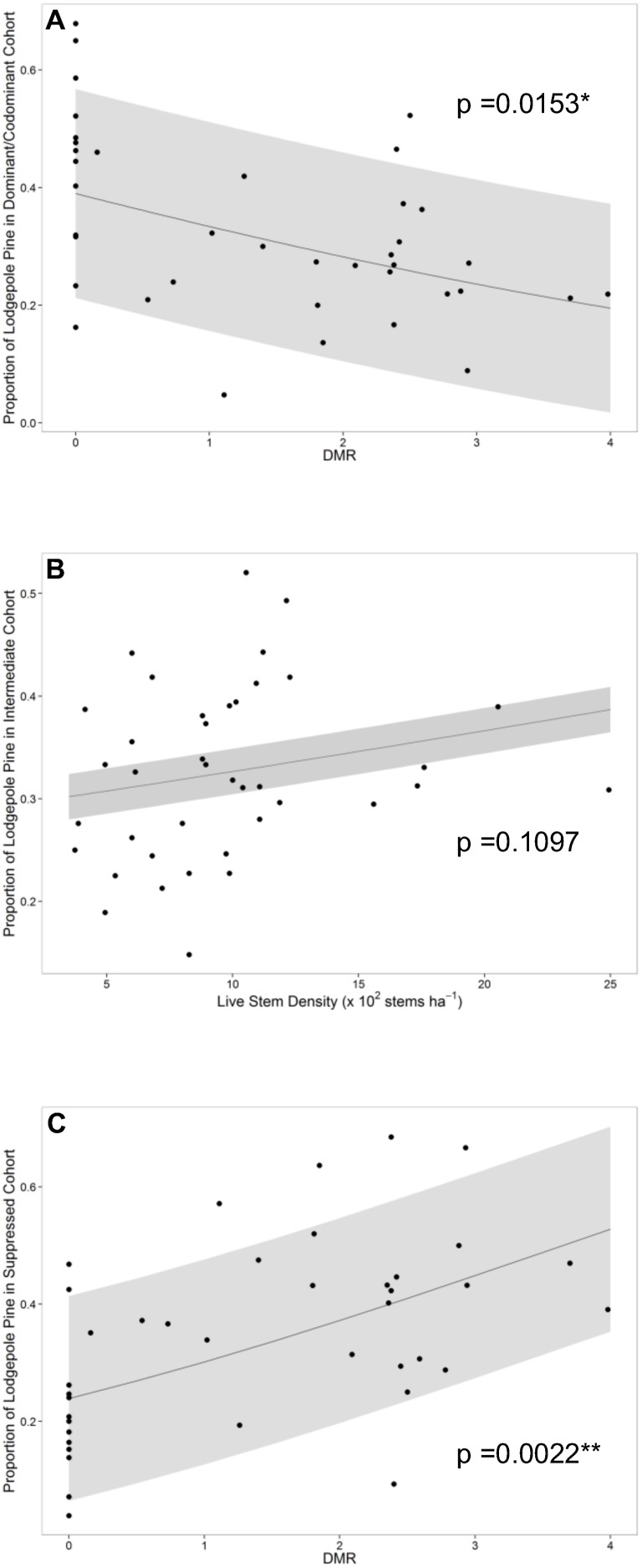
Generalized linear mixed models of proportion of lodgepole pine in cohorts on BIC preferred explanatory variables. Scatterplots of generalized linear mixed models of A) proportion of lodgepole pine in the dominant cohort on dwarf mistletoe rating (DMR), B) proportion of lodgepole pine in the intermediate cohort on stand density and C) proportion of lodgepole pine in the suppressed cohort on DMR with 95% confidence intervals.

The models for the proportion of lodgepole pine in the dominant/codominant cohort and the proportion of lodgepole pine in the suppressed cohort used DMR as their only predictor in the BIC preferred models (Tables S6, S7). There was strong evidence that the proportion of lodgepole pine in the dominant/codominant cohort decreased with DMR severity (Χ^2^_1_ = 5.88, p = 0.0153) while the proportion of lodgepole pine in the suppressed cohort increased with DMR severity (Χ^2^_1_ = 9.35, p = 0.0022) (Table 2, Figure 4). Each doubling of plot-level DMR was associated with a decrease in the odds of a given lodgepole pine tree being in the dominant/codominant cohort by an estimated 15.4% (95% CI: 5.3, 42.2%). Conversely, each doubling of plot-level DMR was associated with an increase in the odds of a lodgepole pine tree being in the suppressed cohort by an estimated 24.6% (95% CI: 14.3, 64.6%). The proportion of lodgepole pine in the intermediate cohort used stand density as its only predictor variable in the BIC preferred model (Table S8). However, there was no evidence of a significant relationship in the preferred model (Χ^2^_1_ = 2.56, p = 0.1097) (Table 2), nor was there evidence of a significant relationship between the proportion of the stand in the intermediate cohort with DMR (Χ^2^_1_ = 0.46, p = 0.4970) (Figure 4).

**Figure 5:**
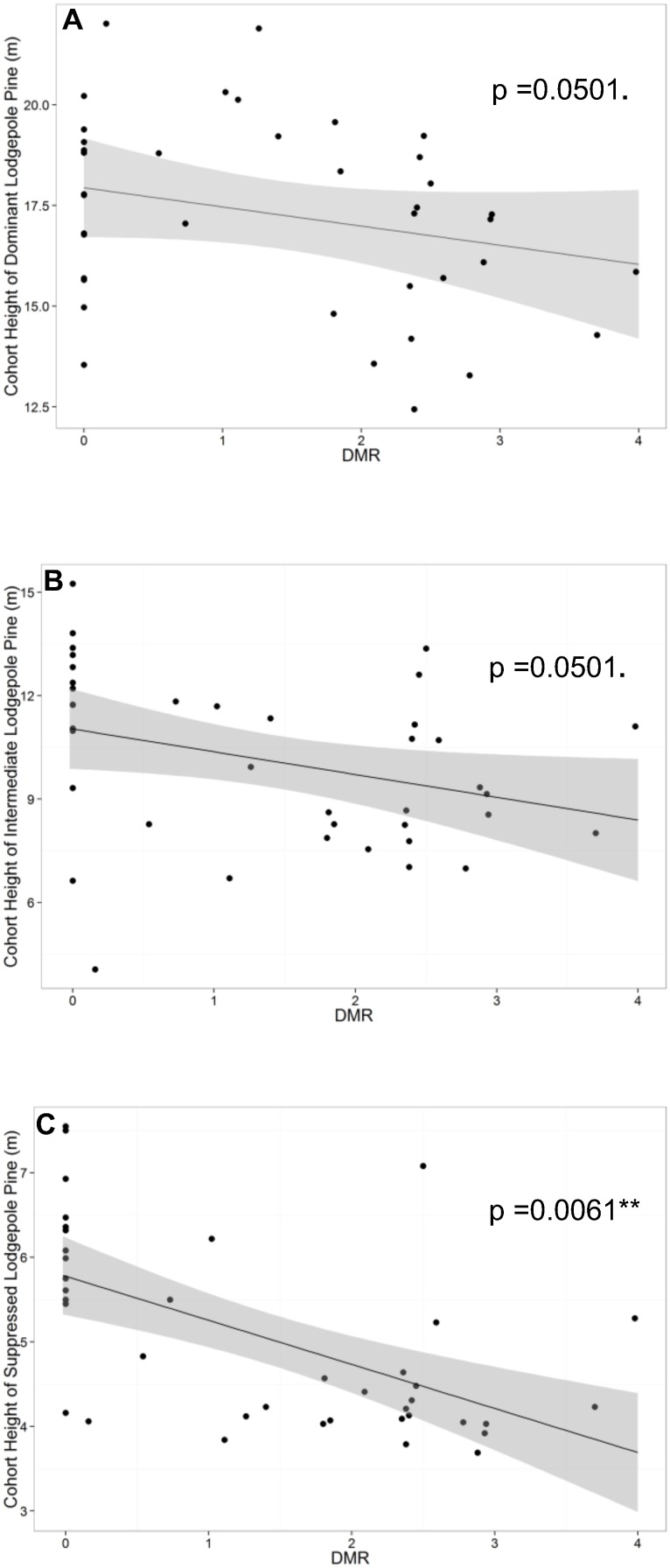
Linear mixed models of cohort height on dwarf mistletoe rating. Scatterplots of linear mixed models of A) cohort height of dominant lodgepole pine (m) with stand density fixed at its mean, B) cohort height of intermediate lodgepole pine (m), and C) cohort height of suppressed lodgepole pine (m) on dwarf mistletoe rating (DMR) with 95% confidence intervals.

Cohort height of intermediates and cohort height of suppressed trees were both best predicted by DMR alone (Tables S9, S10), while the preferred model for cohort height of dominant/codominants included both DMR and stand density as predictors (Table S11). There was suggestive evidence that cohort height of dominant/codominants decreased with DMR after accounting for stand density (Table 2). There was an estimated 0.48 meter decrease (95% CI: 1.13 meter decrease, 0.18 meter increase) in mean dominant/codominant cohort height with each unit increase in DMR, holding stand density at its mean (F_1,24_ = 4.257, p = 0.0501) (Figure 5). Evidence of a relationship of cohort height of intermediates to DMR was also suggestive (F_1,25_ = 4.24, p = 0.0501) (Table 2). There was an estimated 0.66 (95% CI: 0, 1.33) meter decrease in the mean cohort height of intermediate lodgepole pine for each unit increase in DMR (Figure 5). However, there was strong evidence of an effect of DMR on cohort height of suppressed trees (F_1,25_ = 8.975, p = 0.0061) (Table 2). There was an estimated 0.45 (95% CI: 0.14, 0.76) meter decrease in the mean cohort height of suppressed trees for each unit increase in DMR (Figure 5).

**Figure 6:**
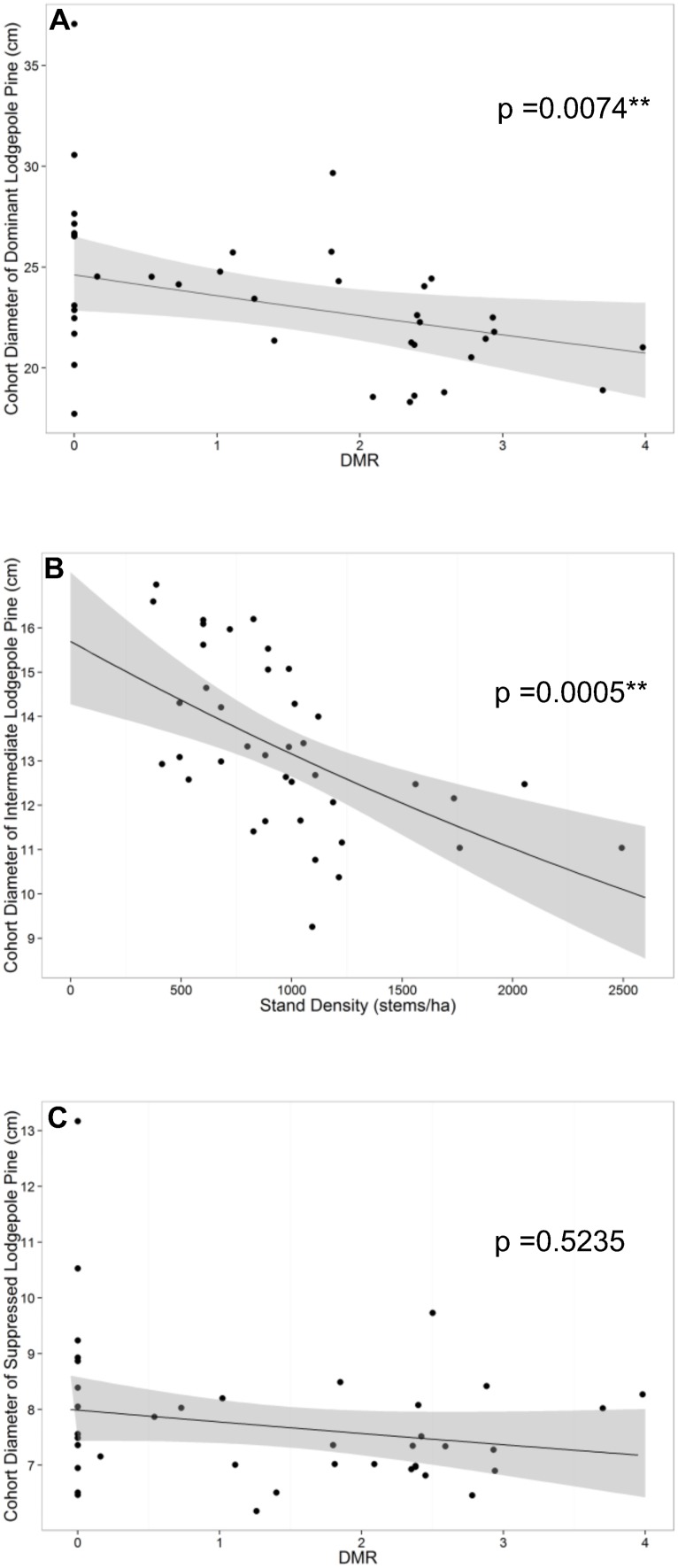
Linear mixed models of log cohort diameter on BIC preferred explanatory variables. Scatterplots of backtransformed linear mixed models of A) natural logarithm of cohort diameter of dominant lodgepole pine (cm) on dwarf mistletoe rating (DMR) with stand density fixed at its mean, B) natural logarithm of cohort diameter of intermediate lodgepole pine (cm) on stand density and C) natural logarithm of cohort diameter of suppressed lodgepole pine on DMR with 95% confidence intervals.

Conversely, there was no evidence of an effect of DMR on the natural logarithm of cohort diameter of suppressed trees (F_1,25_ = 0.419, p = 0.5235) (Table 2, Figure 6). Although other models were equally preferred by BIC, no significant relationships were found within the set of candidate models (Table S12). However, the BIC preferred model (Table S13) showed there was strong evidence of an effect of DMR on the natural logarithm of cohort diameter of dominant/codominants after accounting for stand density (F_1,24_ = 8.563, p = 0.0074) (Table 2). There was an estimated 4.2% (95% CI: 0.3%, 8.0%) decrease in the median cohort diameter of dominant/codominants for each unit increase in DMR after accounting for stand density (Figure 6). The BIC indicated that the natural logarithm of cohort diameter of intermediate trees was best explained by stand density alone (F_1,25_ = 16.212, p = 0.0005) (Table 2, Figure 6), with no evidence of a significant effect of DMR (Table S14).

## Discussion

### Effects of Dwarf Mistletoe on Stand Structure and Cohort Distribution

Dwarf mistletoe is associated with reduced canopy volume, greater representation of the suppressed cohort, reduced representation and average diameter of the dominant/codominant cohort, and reduced average height of the suppressed cohort in lodgepole pine forests. This parasitic plant has a profound influence on the structure of lodgepole pine forests in central Oregon. *Arceuthobium americanum* is common throughout the area, so it is apparent that mountain pine beetle does not eradicate it by killing the host trees. Conversely, it appears that mountain pine beetle activity may exacerbate the stand-level infection by removing the largest trees in the stand and leaving the suppressed, dwarf mistletoe infected trees as described in previous research [10]. Based on our understanding of the epidemiology of *A. americanum* [30], we conclude that dwarf mistletoe then spreads onto the remaining uninfected trees and new regeneration in the stand with increased light availability and space in the mid and lower canopy due to the removal of large trees [61], thereby reducing overall productivity and growth. Although this may be thought of as a negative influence dwarf mistletoe has other ecosystem influences in many forest types including lodgepole pine forests. Dwarf mistletoes provide both food (from the dwarf mistletoe plants themselves) and nesting resources (from the witches’ brooms induced by many dwarf mistletoe species) for numerous bird, mammal, and insect species [30], [62]. The multiple effects of dwarf mistletoes on forest stands indicate that the overall effect of these organisms in a forest is complex and may be viewed as either negative or positive depending on management objectives [63].

The proportion of lodgepole pine in the dominant/codominant cohort significantly decreased as dwarf mistletoe severity increased in plots 21–28 years TSB. Conversely, the proportion of lodgepole pine in the suppressed cohort significantly increased with increased dwarf mistletoe severity. Both findings are consistent with previous work in dwarf mistletoe infected lodgepole pine [34], [36] but our findings are more broadly applicable due to our randomly selected sites and intensive sampling of stands in our study area. The shift in proportion of trees in each cohort indicates that high levels of dwarf mistletoe may keep residual trees in the suppressed cohort after a mountain pine beetle mortality event longer than in stands little or no dwarf mistletoe. However, this shift associated with increased DMR was not reflected in the proportion of trees in the intermediate cohort. This may be attributed to an equivalent proportional shift of both the dominant/codominant cohort and the intermediate cohort to the next smallest size class, reflecting zero net change of the proportion of the stand in the intermediate cohort.

The reduction in cohort height of suppressed trees and the reduced diameter of dominant/codominant trees is consistent with previous work at the individual tree-scale [34], [35], [36], but our findings suggest that this process is also happening at a stand-scale. Increased dwarf mistletoe infection in a stand is associated with increased representation by the suppressed cohort as well as decreased height within all cohorts and decreased diameter within the dominant/codominant cohort. This suggests that the presence of dwarf mistletoe has the ability to slow stand recovery after a mountain beetle epidemic as compared with that of uninfected stands. Although the presence of dwarf mistletoe likely alters the trajectory of stand development, published studies regarding post-mountain pine beetle epidemic stand structure and ecosystem recovery have not accounted for its effects [6], [11], [15]. Dwarf mistletoe is impacting overall stand development post-mountain pine beetle outbreak and its effects should be incorporated to accurately project recovery of stands experiencing mortality.

Total canopy volume indicates the relative amount of available space that is occupied by any given forest. Our data supports the contention that dwarf mistletoe slows the recolonization of available space in these low diversity lodgepole pine ecosystems in the decades following mountain pine beetle mortality. Previous research in central Oregon lodgepole pine showed that the volume of individual crowns were reduced in lodgepole pine trees with increased DMR [39], but sampling occurred in stands in which total canopy volume did not change with infection level [64]. Our results may differ due to our random sampling method and control of previous mountain pine beetle activity, which has not been accounted for in previous work.

Previous work on dwarf mistletoe in central Oregon lodgepole pine has found various results with respect to stand basal area and density, which were not influenced by DMR in this study. One study found no relationship between stand basal area and DMR [36], while another study found a significant reduction in stand basal area associated with increased dwarf mistletoe severity [33]. Both studies found that stand density increases with DMR. The inconsistency of results is likely a result of a disparity in mountain pine beetle legacy, as previous studies of dwarf mistletoe effects did not account for this factor. Previous research has shown that stand basal area and stand density change significantly over time after a mountain pine beetle mortality event [11], [12], [13], [15], which may confound the structural effects of other disturbances. The discrepancy in the understanding of the relationship between dwarf mistletoe and these stand metrics in lodgepole pine forests indicates that interpretation of these relationships must be made within the context of the time since the previous mountain pine beetle epidemic.

### Potential Effects of Structural Heterogeneity Associated with Dwarf Mistletoe

Lodgepole pine dwarf mistletoe’s influences on canopy volume, proportion of the stand in each cohort, and average diameter and height within cohorts show that its presence at various severities on the landscape introduces structural heterogeneity to lodgepole pine forests. Although dwarf mistletoe decreases vigor of individual trees [30], heterogeneity of stand structure leads to higher overall landscape resistance and resilience to various disturbances in many systems [11], [19], [27]. Diverse structure introduced by dwarf mistletoe may actually increase landscape resistance and resilience to disturbances, such as mountain pine beetle epidemics. To reach epidemic populations, mountain pine beetle needs densely stocked dominant and codominant lodgepole pine in which to lay their brood [9]. These conditions are more likely to be found in stands uninfected or lightly infected with dwarf mistletoe, given our results of increased proportion of the suppressed cohort, as well as decreased diameter of the dominant/codominant cohort in stands with severe dwarf mistletoe. Although this study did not directly address landscape-level processes, we hypothesize that heterogeneity of stand structure associated with dwarf mistletoe may influence the pattern and extent of mountain pine beetle mortality on the landscape. We hypothesize that dwarf mistletoe may create patches of mountain pine beetle habitat of varying suitability at a given time. The presence of dwarf mistletoe in lodgepole pine forests post-mountain pine beetle should be addressed at a landscape scape to better understand this relationship.

Furthermore, the relationship between decreased vigor at the individual tree scale associated with dwarf mistletoe and mountain pine beetle susceptibility is poorly understood. Several studies have suggested that during the early phase of an epidemic, trees which have low vigor may be preferred by mountain pine beetle due to lowered defense capabilities [65], [66]. Others have shown that more successful mountain pine beetle brood production occurs in trees with thick phloem [10], [67] which is related to strong tree vigor [68]. This has led to the hypothesis that dwarf mistletoe-infected trees are less susceptible to mountain pine beetle attack because they have thinner phloem than trees without dwarf mistletoe [69], [70], [71]. However, the evidence that dwarf mistletoe infection decreases phloem thickness in lodgepole pine is inconclusive. One study found that infected lodgepole pine have significantly lower phloem thickness than uninfected trees, and concluded that there was a negative relationship between dwarf mistletoe infection and mountain pine beetle brood production [10]. Conversely, another study found no relationship between dwarf mistletoe infection and phloem thickness in lodgepole pine [71]. It is possible that the presence of dwarf mistletoe has some influence on individual trees’ ability to support a mountain pine beetle brood, thereby further intertwining the effects of each disturbance on lodgepole pine forest structure. However, further research is required to discern the nature of this relationship.

An additional factor which confounds the understanding of the ecology of dwarf mistletoe-infected lodgepole pine forests is the effect of mountain pine beetle epidemics on overall stand-level dwarf mistletoe rating. This interaction is not well understood. A study of post-mountain pine beetle dwarf mistletoe severity in British Columbia found a higher level of dwarf mistletoe in unattacked trees than in recently attacked trees, suggesting that mountain pine beetle may prefer uninfected trees to infected trees [72]. They hypothesize that dwarf mistletoe will intensify in stands post-mountain pine beetle epidemic, rather than decrease. We do not have data regarding dwarf mistletoe in these stands pre-mountain pine beetle epidemic, so it is unclear whether or not the epidemic intensified or reduced dwarf mistletoe infection in these stands. The frequency with which we observed dwarf mistletoe in our study area supports the hypothesis that mountain pine beetle epidemics do not remove dwarf mistletoe from attacked stands. Given our understanding of mountain pine beetle behavior, we hypothesize that an epidemic leads to an increase in the stand level DMR due to the removal of the largest and most vigorous trees, while leaving the dwarf mistletoe infected and suppressed trees. However, further research regarding host selection by mountain pine beetle and survival of attacked hosts is necessary to fully understand this relationship in lodgepole pine.

## Conclusions

The mountain pine beetle influences many stand attributes, so it is important to interpret structural effects within the context of the previous mountain pine beetle epidemic. Conversely, when attempting to understand the influence of mountain pine beetle on stand structure and ecosystem processes in lodgepole pine, it is imperative to incorporate dwarf mistletoe effects. Structural differences associated with dwarf mistletoe create heterogeneous structure in this forest type and may have a significant influence on the productivity, resistance, and resilience of these stands in both positive and negative ways. Research at multiple spatial and temporal scales should be conducted to understand the complexity of the disturbance ecology of lodgepole pine forests. Furthermore, dwarf mistletoe may be similarly influencing ecosystem structure and function of other forest types and investigations of disturbance ecology should include dwarf mistletoes where they occur.

## Supporting Information

Table S1 **Plot locations.**
(DOCX)

Table S2 **Cohort characteristics of 39 lodgepole pine plots within 13 stands in the Deschutes National Forest, Oregon.**
(DOCX)

Table S3 **BIC table for the natural logarithm of stand density model.**
(DOCX)

Table S4 **BIC table for stand basal area model.**
(DOCX)

Table S5 **BIC table for the natural logarithm of canopy volume model.**
(DOCX)

Table S6 **BIC table for the proportion of lodgepole pine in the dominant/codominant cohort model.**
(DOCX)

Table S7 **BIC table for the proportion of lodgepole pine in the suppressed cohort model.**
(DOCX)

Table S8 **BIC table for the proportion of lodgepole pine in the intermediate cohort model.**
(DOCX)

Table S9 **BIC table for the cohort height of intermediates model.**
(DOCX)

Table S10 **BIC table for the cohort height of suppressed model.**
(DOCX)

Table S11 **BIC table for the cohort height of dominant/codominants model.**
(DOCX)

Table S12 **BIC table for the natural logarithm of cohort diameter of suppressed model.**
(DOCX)

Table S13 **BIC table for the natural logarithm of cohort diameter of dominant/codominants model.**
(DOCX)

Table S14 **BIC table for the natural logarithm of cohort diameter of intermediates model.**
(DOCX)

## References

[1] Lotan JE, Critchfield WB (1990) *Pinus contorta* (Dougl. ex. Loud.) lodgepole pine. In: Burns RM, Honkala BH, technical coordinators. Silvics of North America. Volume 1. Conifers. USDA Agricultural Handbook 654. Washington, DC, USA. 302–315.

[2] Raffa KF, Aukema BH, Bentz BJ, Carroll AL, Hicke JA, et al (2008) Cross-scale drivers of natural disturbances prone to anthropogenic amplification: the dynamics of bark beetle eruptions. Bioscience 58(6): 501–517.

[3] Bright BC, Hicke JA, Hudak AT (2012) Landscape-scale analysis of aboveground tree carbon stocks affected by mountain pine beetles in Idaho. Environ Res Lett 7(4): 6.

[4] Mikkelson KM, Bearup LA, Maxwell RM, Stednick JD, McCray JE, et al (2013) Bark beetle infestation impacts on nutrient cycling, water quality and interdependent hydrological effects. Biogeochemistry 115: 1–21.

[5] Hicke JA, Johnson MC, Hayes JL, Preisler HK (2012) Effects of bark beetle-caused tree mortality on wildfire. For Ecol Manage 271: 81–90.

[6] Kayes LJ, Tinker DB (2012) Forest structure and regeneration following a mountain pine beetle epidemic in southeastern Wyoming. For Ecol Manage 263: 57–66.

[7] Mitchell RG, Preisler HK (1998) Fall rate of lodgepole pine killed by the mountain pine beetle in central Oregon. West J Appl For 13(1): 23–26.

[8] Simard M, Romme WH, Griffin JM, Turner MG (2011) Do mountain pine beetle outbreaks change the probability of active crown fire in lodgepole pine forests? Ecol Monogr 81(1): 3–24.

[9] Amman GD (1977) The role of the mountain pine beetle in lodgepole pine ecosystems: impact on succession. In: Mattson WJ, editor. The role of arthropods in forest ecosystems: Springer-Verlag. 3–8.

[10] Roe AL, Amman GD (1970) The mountain pine beetle in lodgepole pine forests. USDA Forest Service Research Paper INT-71. Rocky Mountain Research Station, Ogden, Utah, USA.

[11] Diskin M, Rocca ME, Nelson KN, Aoki CF, Romme WH (2011) Forest developmental trajectories in mountain pine beetle disturbed forests of Rocky Mountain National Park, Colorado. Can J For Res 41(4): 782–792.

[12] Klutsch JG, Negrón JF, Costello SL, Rhoades CC, West DR, et al (2009) Stand characteristics and downed woody debris accumulations associated with a mountain pine beetle (*Dendroctonus ponderosae* Hopkins) outbreak in Colorado. For Ecol Manage 258(5): 641–649.

[13] Pelz KA, Smith FW (2012) Thirty year change in lodgepole and lodgepole/mixed conifer forest structure following 1980s mountain pine beetle outbreak in western Colorado, USA. For Ecol Manage 280: 93–102.

[14] Sibold JS, Veblen TT, Chipko K, Lawson L, Mathis E, et al (2007) Influences of secondary disturbances on lodgepole pine stand development in Rocky Mountain National Park. Ecol Appl 17(6): 1638–1655. 17913129 10.1890/06-0907.1

[15] Collins BJ, Rhoades CC, Hubbard RM, Battaglia MA (2011) Tree regeneration and future stand development after bark beetle infestation and harvesting in Colorado lodgepole pine stands. For Ecol Manage 261(11): 2168–2175.

[16] Varhola A, Coops NC, Bater CW, Teti P, Boon S, et al (2010) The influence of ground- and lidar-derived forest structure metrics on snow accumulation and ablation in disturbed forests. Can J For Res 40(4): 812–821.

[17] Koch AJ, Drever MC, Martin K (2011) The efficacy of common species as indicators: avian responses to disturbance in British Columbia, Canada. Biodivers Conserv 20(14): 3555–3575.

[18] Mainwaring DB, Maguire DA (2004) The effect of local stand structure on growth and growth efficiency in heterogeneous stands of ponderosa pine and lodgepole pine in central Oregon. Can J For Res 34(11): 2217–2229.

[19] Astrup RK, Coates D, Hall E (2008) Recruitment limitation in forests: lessons from an unprecedented mountain pine beetle epidemic. For Ecol Manage 256(10): 1743–1750.

[20] Klutsch JG, Battaglia MA, West DR, Costello SL, Negrón JF (2011) Evaluating potential fire behavior in lodgepole pin-dominated forests after a mountain pine beetle epidemic in north-central Colorado. West J Appl For 26(3): 101–109.

[21] Geiszler DR, Gara RI, Driver CH, Gallucci VF, Martin RE (1980) Fire, fungi, and beetle influences on a lodgepole pine ecosystem of south-central Oregon. Oecologia 46(2): 239–243. 28309679 10.1007/BF00540132

[22] Smith GD, Carroll AL, Lindgren BS (2011) Facilitation in bark beetles: endemic mountain pine beetle gets a helping hand. Agric For Entomol 13(1): 37–43.

[23] Paine RT, Tegner MJ, Johnson EA (1998) Compounded perturbations yield ecological surprises. Ecosystems 1(6): 535–545.

[24] D’Amato AW, Fraver S, Palik BJ, Bradford JB, Patty L (2011) Singular and interactive effects of blowdown, salvage logging, and wildfire in sub-boreal pine systems. For Ecol Manage 262(11): 2070–2078.

[25] Buma B, Wessman CA (2012) Differential species responses to compounded perturbations and implications for landscape heterogeneity and resilience. For Ecol Manage 266: 25–33.

[26] Seidl R, Fernandes PM, Fonseca TF, Gillet F, Jönsson AM, et al (2011) Modelling natural disturbances in forest ecosystems: a review. Ecol Model 222: 903–924.

[27] Turner MG (2010) Disturbance and landscape dynamics in a changing world. Ecology 91(10): 2833–2849. 21058545 10.1890/10-0097.1

[28] Hull RJ, Leonard OA (1964a) Physiological aspects of parasitism in mistletoes (*Arceuthobium* and *Phoradendron*). I. The carbohydrate nutrition of mistletoe. Plant Physiol 39(6): 996–1007. 16656050 10.1104/pp.39.6.996 PMC550208

[29] Hull RJ, Leonard OA (1964b) Physiological aspects of parasitism in mistletoes (*Arceuthobium* and *Phoradendron*). II. The photosynthetic capacity of mistletoe. Plant Physiol 39(6): 1008–1017. 16656016 10.1104/pp.39.6.1008 PMC550209

[30] Hawksworth FG, Wiens D (1996) Dwarf mistletoes: biology, pathology, and systematics. USDA Forest Service Agricultural Handbook 709, Fort Collins, Colorado, USA.

[31] Johnson DW, Hawksworth FG, Drummond DB (1981) Yield loss of lodgepole pine stands to dwarf mistletoe in Colorado and Wyoming national forests. Plant Dis 65: 437–438.

[32] Mathiasen RL, Nickrent DL, Shaw DC, Watson DM (2008) Mistletoes: pathology, systematics, ecology, and management. Plant Dis 92(7): 988–1006. 10.1094/PDIS-92-7-0988 30769529

[33] Godfree RC, Tinnin RO, Forbes RB (2002a) The effects of dwarf mistletoe, witches’ brooms, stand structure, and site characteristics on the crown architecture of lodgepole pine in Oregon. Can J For Res 32(8): 1360–1371.

[34] Hawksworth FG, Hinds TE (1964) Effects of dwarfmistletoe on immature lodgepole pine stands in Colorado. J Forest 62: 27–32.

[35] Baranyay JA, Safranyik L (1970) Effect of dwarf mistletoe on growth and mortality of lodgepole pine stands in Alberta. Canadian Forestry Service, Department of Fisheries and Forestry Publication 1285. Ottawa, Ontario, Canada. 19 p.

[36] Wanner JL, Tinnin RO (1989) Some effects of infection by *Arceuthobium americanum* on the population dynamics of *Pinus contorta* in Oregon. Can J For Res 19: 736–742.

[37] Weir JR (1916) Mistletoe injury to conifers in the Northwest. U.S. Department of Agriculture Bulletin 306. Washington, DC., USA.

[38] Wanner JL (1986) Effects of infection by dwarf mistletoe (*Arceuthobium americanum*) on the population dynamics of lodgepole pine (*Pinus contora*). Ph.D. dissertation, Portland State University, Portland, OR.

[39] Godfree RC, Tinnin RO, Forbes RB (2002b) Relationships between *Arceuthobium americanum* and the structure of *Pinus contorta* var. *murrayana* stands in central Oregon. Plant Ecol 165(1): 69–84.

[40] Klutsch JG, Beam RD, Jacobi WR, Negrón JF (2014) Bark beetles and dwarf mistletoe interact to alter downed woody material, canopy structure, and stand characteristics in northern Colorado ponderosa pine. For Ecol Manage 315(1): 63–71.

[41] Simpson M (2007) Forested plant associations of the Oregon East Cascades. USDA Forest Service Technical Paper R6-NR-ECOL-TP-03-2007.

[42] Simard M, Powell EN, Raffa KF, Turner MG (2012) What explains landscape patterns of tree mortality caused by bark beetle outbreaks in Greater Yellowstone? Global Ecol Biogeogr 21: 556–567.

[43] Hansen EM (2014) Forest development and carbon dynamics after mountain pine beetle outbreaks. Forest Sci 60(3): 476–488.

[44] Axelson JN, Alfaro RI, Hawkes BC (2009) Influence of fire and mountain pine beetle on the dynamics of lodgepole pine stands in British Columbia, Canada. For Ecol Manage 257(9): 1874–1882.

[45] Axelson JN, Alfaro RI, Hawkes BC (2010) Changes in stand structure in uneven-aged lodgepole pine stands impacted by mountain pine beetle epidemics and fires in central British Columbia. Forest Chron 86(1): 87–99.

[46] Volland LA (1988) Plant associations of the central Oregon pumice zone. USDA Forest Service Technical Paper R6-ECOL-104-1985.

[47] Franklin JF, Dyrness CT (1973) Natural vegetation of Oregon and Washington. USDA Forest Service General Technical Report PNW-GTR-008. Pacific Northwest Research Station, Portland, Oregon, USA. 427 p.

[48] Western Regional Climate Center [WRCC] (2013) Monthly precipitation, minimum and maximum temperatures for Wickiup Dam climate station. Available: http://www.wrcc.dri.edu/. Accessed 2013 Jan 6.

[49] USDA Forest Service [USFS] (2012) Forest insect and disease aerial survey data, Pacific Northwest region. Available: http://www.fs.usda.gov/detail/r6/forest-grasslandhealth/insects-diseases/. Accessed 2012 Feb 21.

[50] Stevens DL, Olsen AR (2004) Spatially balanced sampling of natural resources. J Am Stat Assoc 99(465): 262–278.

[51] ESRI (2007) ArcGIS 9.3. Environmental Systems Research Institute, Redlands, California, USA.

[52] Van Pelt R, Nadkarni NM (2004) Development of canopy structure in *Pseudotsuga menziesii* forests in the southern Washington Cascades. Forest Sci 50(3): 326–341.

[53] Hawksworth FG (1977) The 6-class dwarf mistletoe rating system. USDA Forest Service General Technical Report RM-48, Rocky Mountain Research Station, Fort Collins, Colorado, USA.

[54] Shaw DC, Freeman EA, Mathiasen RL (2000) Evaluating the accuracy of ground-based hemlock dwarf mistletoe rating: a case study using the Wind River Canopy Crane. West J Appl For 15(1): 8–14.

[55] Coder KD (2000) Crown shape factors & volumes. Tree biomechanics series. Extension publication FOR00-32. University of Georgia, Warnell School of Forest Resources, Athens, Georgia, USA.

[56] Burnham KP, Anderson DR (2002) Model selection and multimodel inference: a practical information-theoretic approach. Second edition. New York City: Springer.

[57] Ramsey FL, Schafer DW (2013) The Statistical Sleuth: A course in methods of data analysis. Third edition. Boston: Brooks/Cole.

[58] Browne WJ, Subramanian SV, Jones K, Goldstein H (2005) Variance partitioning in multilevel logistic models that exhibit overdispersion. J Roy Stat Soc A Sta 168(3): 599–613.

[59] Nakagawa S, Schielzeth H (2013) A general and simple method for obtaining R^2^ from generalized linear mixed-effects models. Methods in Ecol Evol 4(2): 133–142.

[60] R Development Core Team (2009) R: A Language and Environment for Statistical Computing. Version 2.12.0. R Foundation for Statistical Computing, Vienna, Austria. Available: http://www.R-project.org.

[61] Parmeter JR (1978) Forest stand dynamics and ecological factors in relation to dwarf mistletoe spread, impact, and control. In: Scharpf RF, Parmeter JR editors. Proceedings of the symposium on dwarf mistletoe control through forest management. 11–13 April 1978, Berkeley, California, USA. 16–30.

[62] Shaw DC, Watson DM, RL Mathiasen (2004) Comparison of dwarf mistletoes (*Arceuthobium* spp., Viscaceae) in the western United States with mistletoes (*Amyema* spp., Loranthaceae) in Australia–ecological analogs and reciprocal models for ecosystem management. Aust J Bot 52(4): 481–498.

[63] Winder RS, Shamoun SF (2006) Forest pathogens: friend or foe to biodiversity? Can J Plant Pathol 18: S221–S227.

[64] Godfree RC, Tinnin RO, Forbes RB (2003) Relationships between dwarf mistletoe and the canopy structure of an old-growth lodgepole pine forest in central Oregon. Can J For Res 33: 997–1009.

[65] Kulakowski D, Jarvis D (2013) Low-severity fires increase susceptibility of lodgepole pine to mountain pine beetle outbreaks in Colorado. For Ecol Manage 289: 544–550.

[66] Amman GD (1972) Mountain pine beetle brood production in relation to thickness of lodgepole pine phloem. J Econ Entomol 65(1): 138–140.

[67] Cole DM (1973) Estimation of phloem thickness in lodgepole pine. USDA Forest Service Research Paper INT-148, Ogden, Utah, USA. 10 p.

[68] Amman GD (1978) Biology, ecology, and causes of outbreaks of the mountain pine beetle in lodgepole pine forests. In: Kibbee DL, Berryman AA, Amman GD, Stark RW, editors. Theory and practice of mountain pine beetle management in lodgepole pine forests: proceedings of the symposium. 25–27 April 1978, Washington State University, Pullman, WA, USA. 39–53.

[69] McGregor MD (1978) Management of mountain pine beetle in lodgepole pine stands in the Rocky Mountain area. In: Kibbee DL, Berryman AA, Amman GD, Stark RW, editors. Theory and practice of mountain pine beetle management in lodgepole pine forests: proceedings of the symposium. 25–27 April 1978, Washington State University, Pullman, WA, USA. 129–139.

[70] Stevens RE, Hawksworth FG (1984) Insect-dwarf mistletoe associations: an update. In: Hawksworth FG, Scharpf RF, technical coordinators. Biology of dwarf mistletoes: proceedings of the symposium. 8 August 1984, Colorado State University, Fort Collins, Colorado, USA. 94–101.

[71] Hawksworth FG, Lister CK, Cahill DB (1983) Phloem thickness in lodgepole pine: its relationship to dwarf mistletoe and mountain pine beetle (Coleoptera: Scolytidae). Environ Entomol 12: 1447–1448.

[72] Shore TL, Andrews RJ, van Sickle GA (1982) Survey for dwarf mistletoe infection in lodgepole pine regeneration under mountain pine beetle-attacked stands in the Chilcotin. Special Report. Environment Canada, Canadian Forestry Service, Victoria, British Columbia, Canada.

