# Supplementary material for: Effects of Dwarf Mistletoe on Stand Structure of Lodgepole Pine Forests 21-28 Years Post-Mountain Pine Beetle Epidemic in Central Oregon
Source: PLoS One. 2014 Sep 15;9(9):e107532. doi: 10.1371/journal.pone.0107532 (PMC4164639; doi:10.1371/journal.pone.0107532)
Supplement: Table S1 — Plot locations. (DOCX) [file pone.0107532.s001.docx]

**Table S1.** Plot locations.

| **Stand** | **Plot** | **Easting (0 m)** | **Northing**  **(0 m)** | **Easting**  **(75 m)** | **Northing (75 m)** |
| --- | --- | --- | --- | --- | --- |
|  | 1 | 582777 | 4820232 | 582846 | 4820222 |
| CRL | 2 | 582796 | 4820548 | 582859 | 4820575 |
|  | 3 | 582996 | 4820409 | 583066 | 4820416 |
|  | 1 | 596218 | 4851266 | 596266 | 4851333 |
| CRP | 2 | 596327 | 4851514 | 596372 | 4851569 |
|  | 3 | 596215 | 4851222 | 596220 | 4851155 |
|  | 1 | 606907 | 4853611 | 606950 | 4853661 |
| CRP2 | 2 | 606865 | 4853612 | 606802 | 4853648 |
|  | 3 | 606872 | 4853547 | 606942 | 4853557 |
|  | 1 | 593854 | 4859463 | 593921 | 4859495 |
| CUL2 | 2 | 593955 | 4859181 | 593881 | 4859191 |
|  | 3 | 593536 | 4858851 | 593609 | 4858849 |
|  | 1 | 595992 | 4858228 | 596036 | 4858284 |
| CUL6 | 2 | 596365 | 4858190 | 596444 | 4858201 |
|  | 3 | 596310 | 4858278 | 596253 | 4858330 |
|  | 1 | 616618 | 4851072 | 616659 | 4851138 |
| DES | 2 | 616691 | 4850868 | 616621 | 4850895 |
|  | 3 | 616763 | 4851136 | 616839 | 4851136 |
|  | 1 | 667098 | 4841202 | 667031 | 4841178 |
| EFR | 2 | 667003 | 4841299 | 666949 | 4841351 |
|  | 3 | 667331 | 4841469 | 667272 | 4841425 |
|  | 1 | 665659 | 4836181 | 665728 | 4836204 |
| EFR3 | 2 | 666081 | 4836692 | 666150 | 4836682 |
|  | 3 | 666122 | 4836343 | 666193 | 4836342 |
|  | 1 | 595913 | 4801544 | 595923 | 4801619 |
| LDES | 2 | 595867 | 4801776 | 595917 | 4801829 |
|  | 3 | 595796 | 4801767 | 595840 | 4801831 |
|  | 1 | 598559 | 4861559 | 598530 | 4861631 |
| LVLK | 2 | 599125 | 4861229 | 599188 | 4861190 |
|  | 3 | 598111 | 4862305 | 598191 | 4862293 |
|  | 1 | 584091 | 4826802 | 584058 | 4826740 |
| ODL | 2 | 583762 | 4826714 | 583835 | 4826709 |
|  | 3 | 583692 | 4826804 | 583760 | 4826768 |
|  | 1 | 641092 | 4839924 | 641097 | 4839852 |
| PAU | 2 | 641399 | 4840588 | 641428 | 4840516 |
|  | 3 | 640929 | 4840448 | 641000 | 4840440 |
|  | 1 | 598950 | 4855963 | 598884 | 4855995 |
| SNC | 2 | 599033 | 4855931 | 599107 | 4855932 |
|  | 3 | 599054 | 4855889 | 599088 | 4855824 |

Note: Easting and Northing = GPS coordinates in UTM (taken at each end of the belt transect)
