# Supplementary material for: Effects of Dwarf Mistletoe on Stand Structure of Lodgepole Pine Forests 21-28 Years Post-Mountain Pine Beetle Epidemic in Central Oregon
Source: PLoS One. 2014 Sep 15;9(9):e107532. doi: 10.1371/journal.pone.0107532 (PMC4164639; doi:10.1371/journal.pone.0107532)
Supplement: Table S2 — Cohort characteristics of 39 lodgepole pine plots within 13 stands in the Deschutes National Forest, Oregon. (DOCX) [file pone.0107532.s002.docx]

**Table S2.** Cohort characteristics of 39 lodgepole pine plots within 13 stands in the Deschutes National Forest, Oregon.

|  |  |  | **Proportion of Stand in Cohort** | | | **Cohort Height (m)** | | | **Cohort Diameter (cm)** | | |
| --- | --- | --- | --- | --- | --- | --- | --- | --- | --- | --- | --- |
| **Stand** | **Plot** | **DMR** | **Dom** | **Int** | **Sup** | **Dom** | **Int** | **Sup** | **Dom** | **Int** | **Sup** |
| CRL | 1 | 2.78 | 22 | 49 | 29 | 13.3 | 7.0 | 4.1 | 20.5 | 10.4 | 6.5 |
|  | 2 | 2.93 | 9 | 24 | 67 | 17.2 | 9.2 | 3.9 | 22.5 | 14.2 | 7.3 |
|  | 3 | 2.40 | 47 | 44 | 9 | 17.5 | 10.8 | 4.1 | 22.6 | 16.2 | 8.1 |
| CRP | 1 | 0 | 32 | 44 | 24 | 18.9 | 13.8 | 6.9 | 22.5 | 14.0 | 7.5 |
|  | 2 | 1.02 | 32 | 34 | 34 | 20.3 | 11.7 | 6.2 | 24.8 | 13.1 | 8.2 |
|  | 3 | 0 | 65 | 31 | 4 | 19.4 | 15.3 | 7.6 | 21.7 | 12.7 | 8.9 |
| CRP2 | 1 | 1.81 | 20 | 28 | 52 | 19.6 | 8.6 | 4.6 | 29.7 | 10.8 | 7.0 |
|  | 2 | 2.45 | 37 | 33 | 29 | 19.2 | 12.6 | 4.5 | 24.1 | 15.5 | 6.8 |
|  | 3 | 2.94 | 27 | 30 | 43 | 17.3 | 8.6 | 4.0 | 21.8 | 12.1 | 6.9 |
| CUL2 | 1 | 2.42 | 31 | 25 | 45 | 18.7 | 11.2 | 4.3 | 22.3 | 12.6 | 7.5 |
|  | 2 | 2.35 | 26 | 31 | 43 | 15.5 | 8.3 | 4.1 | 18.3 | 11.7 | 6.9 |
|  | 3 | 2.38 | 17 | 15 | 69 | 17.3 | 7.0 | 3.8 | 21.2 | 11.4 | 7.0 |
| CUL6 | 1 | 3.98 | 22 | 39 | 39 | 15.9 | 11.1 | 5.3 | 21.0 | 13.3 | 8.3 |
|  | 2 | 3.70 | 21 | 32 | 47 | 14.3 | 8.0 | 4.2 | 18.9 | 12.5 | 8.0 |
|  | 3 | 2.59 | 36 | 33 | 31 | 15.7 | 10.7 | 5.2 | 18.8 | 11.0 | 7.3 |
| DES | 1 | 0 | 48 | 26 | 26 | 19.1 | 13.2 | 7.5 | 27.7 | 16.1 | 13.2 |
|  | 2 | 0 | 32 | 21 | 47 | 17.8 | 12.8 | 5.6 | 27.2 | 16.0 | 8.4 |
|  | 3 | 0 | 48 | 33 | 18 | 16.8 | 9.3 | 5.5 | 30.6 | 13.1 | 8.1 |
| EFR | 1 | 0 | 44 | 36 | 20 | 16.8 | 11.1 | 6.5 | 26.7 | 15.6 | 8.9 |
|  | 2 | 0 | 59 | 28 | 14 | 18.8 | 12.4 | 5.8 | 37.1 | 17.0 | 10.5 |
|  | 3 | 0 | 46 | 37 | 16 | 15.7 | 11.7 | 6.4 | 23.1 | 15.1 | 9.2 |
| EFR3 | 1 | 0 | 52 | 33 | 15 | 15.0 | 11.0 | 6.0 | 22.9 | 14.7 | 7. 6 |
|  | 2 | 0 | 68 | 25 | 7 | 17.8 | 13.4 | 5.5 | 26.6 | 16.6 | 7.0 |
|  | 3 | 0 | 40 | 39 | 21 | 15.7 | 11.7 | 6.3 | 20.2 | 12.5 | 7.4 |
| LDES | 1 | 0.54 | 21 | 42 | 37 | 18.8 | 8.3 | 4.8 | 24.5 | 13.0 | 7.9 |
|  | 2 | 0 | 23 | 52 | 25 | 20.2 | 12.2 | 6.1 | 26.5 | 13.4 | 6.5 |
|  | 3 | 0.73 | 24 | 39 | 37 | 17.1 | 11.8 | 5.5 | 24.2 | 14.3 | 8.0 |
| LVLK | 1 | 1.11 | 5 | 38 | 57 | 20.1 | 6.7 | 3.8 | 25.7 | 11.6 | 7.0 |
|  | 2 | 2.09 | 27 | 42 | 31 | 13.6 | 7.6 | 4.4 | 18.6 | 11.2 | 7.0 |
|  | 3 | 0 | 16 | 41 | 43 | 13.5 | 6.6 | 4.2 | 17.7 | 9.3 | 6.5 |
| ODL | 1 | 2.50 | 52 | 23 | 25 | 18.1 | 13.4 | 7.1 | 24.4 | 16.2 | 9.7 |
|  | 2 | 1.85 | 14 | 23 | 64 | 18.4 | 8.3 | 4.1 | 24.3 | 15.1 | 8.5 |
|  | 3 | 2.88 | 22 | 28 | 50 | 16.1 | 9.3 | 3.7 | 21.5 | 13.3 | 8.4 |
| PAU | 1 | 2.36 | 29 | 31 | 40 | 14.2 | 8.7 | 4.6 | 21.3 | 12.2 | 7.4 |
|  | 2 | 2.38 | 27 | 31 | 42 | 12.4 | 7.8 | 4.2 | 18.6 | 11.0 | 7.0 |
|  | 3 | 1.80 | 27 | 29 | 43 | 14.8 | 7.9 | 4.0 | 25.8 | 12.5 | 7.4 |
| SNC | 1 | 1.40 | 30 | 23 | 48 | 19.2 | 11.3 | 4.2 | 21.4 | 12.6 | 6.5 |
|  | 2 | 1.26 | 42 | 39 | 19 | 21.9 | 9.9 | 4.1 | 23.4 | 12.9 | 6.2 |
|  | 3 | 0.16 | 46 | 19 | 35 | 21.4 | 8.6 | 4.1 | 24.0 | 14.3 | 7.2 |

Note: Dom = dominant/codominant cohort; Int = intermediate cohort; Sup = suppressed cohort.
