# Supplementary material for: Effects of Dwarf Mistletoe on Stand Structure of Lodgepole Pine Forests 21-28 Years Post-Mountain Pine Beetle Epidemic in Central Oregon
Source: PLoS One. 2014 Sep 15;9(9):e107532. doi: 10.1371/journal.pone.0107532 (PMC4164639; doi:10.1371/journal.pone.0107532)
Supplement: Table S4 — BIC table for stand basal area model. (DOCX) [file pone.0107532.s004.docx]

**Table S4.** BIC table for stand basal area model.

| **Model** | **df** | **BIC** | **ΔBIC** | **BIC weight** | **Evidence ratio** |
| --- | --- | --- | --- | --- | --- |
| ***SBA_ij_ = β_0_ + b_j_ + β_1_DMR_ij_ + ε_ij_*** | 4 | 53.52 | 0 | 0.01 | 1 |
| ***SBA_ij_ = β_0_ + b_j_ + β_1_MPBMORT.L_ij_ + β_2_MPBMORT.M_ij_ + ε_ij_*** | 5 | 55.32 | 1.80 | 5.82E-03 | 2.46 |
| ***SBA_ij_ = β_0_ + b_j_ + β_1_DMR_ij_ + β_2_MPBMORT.L_ij_ + β_3_MPBMORT.M_ij_ + ε_ij_*** | 6 | 57.88 | 4.36 | 1.62E-03 | 8.86 |
| ***SBA_ij_ = β_0_ + b_j_ + β_1_PROD.L_ij_ + β_2_PROD.M_ij_ + ε_ij_*** | 5 | 60.06 | 6.54 | 5.46E-04 | 26.25 |
| ***SBA_ij_ = β_0_ + b_j_ + β_1_DMR_ij_ + β_2_PROD.L_ij_ + β_3_PROD.M_ij_ + ε_ij_*** | 6 | 60.69 | 7.17 | 3.98E-04 | 36.01 |
| ***SBA_ij_ = β_0_ + b_j_ + β_1_DMR_ij_ + β_2_MPBMORT.L_ij_ + β_3_MPBMORT.M_ij_ + β_4_DMR*MPBMORT.L_ij_ + β_5_DMR*MPBMORT.M_ij_ + ε_ij_*** | 8 | 61.64 | 8.12 | 2.47E-04 | 58.07 |
| ***SBA_ij_ = β_0_ + b_j_ + β_1_DMR_ij_ + β_2_MPBMORT.L_ij_ + β_3_MPBMORT.M_ij_ + β_4_PROD.L_ij_ + β_5_PROD.L_ij_ + ε_ij_*** | 8 | 64.83 | 11.31 | 5.02E-05 | 285.32 |
| ***SBA_ij_ = β_0_ + b_j_ + β_1_DMR_ij_ + β_2_PROD.L_ij_ + β_3_PROD.M_ij_ + β_4_DMR*PROD.L_ij_ + β_5_DMR*PROD.M_ij_ + ε_ij_*** | 8 | 64.99 | 11.46 | 4.65E-05 | 308.51 |
| ***SBA_ij_ = β_0_ + b_j_ + β_1_DMR_ij_ + β_2_MPBMORT.L_ij_ + β_3_MPBMORT.M_ij_ + β_4_PROD.L_ij_ + β_5_PROD.M_ij_ + β_6_PROD.L*DMR_ij_ + β_7_PROD.M*DMR_ij_ +β_8_MPBMORT.L*DMR_ij_ + β_9_MPBMORT.M*DMR_ij_ + ε_ij_*** | 12 | 72.54 | 19.02 | 1.06E-06 | 13472.60 |

Note: df= degrees of freedom; BIC = Bayesian Information Criterion; ΔBIC = difference in BIC value as compared with that of the preferred model; *SBA_ij_* = stand basal area of the *ith* stand within the *jth* site; *β_0_* = mean of stand basal area when all additional *β’*s = 0; *DMR*_ij_ = dwarf mistletoe rating of the *ith* stand within the *jth* site; *PROD.L_ij_* = indicator which = 1 when the productivity of the *ith* stand within the *jth* site is low and 0 otherwise; *PROD.M_ij_* = indicator which = 1 when the productivity of the *ith* stand within the *jth* site is moderate and 0 otherwise; *MPBMORT.L_ij_* = indicator which = 1 when the mortality density of the previous mountain pine beetle epidemic of the *ith* stand within the *jth* site is low and 0 otherwise; *MPBMORT.L_ij_* = indicator which = 1 when the mortality density of the previous mountain pine beetle epidemic of the *ith* stand within the *jth* site is moderate and 0 otherwise; *b_j_* = random error for the *jth* site, *b_j_* ~ N(0, σ_b_^2^) and *b_j_* and *b_j’_* are independent; *ε_ij_* = random error from stand basal area measurements *ith* stand replicate within the *jth* site, *ε_ij_* ~ N(0, σ^2^) and *ε_ij_* and ***ε_i’j’_*** are independent.
