# Supplementary material for: Effects of Dwarf Mistletoe on Stand Structure of Lodgepole Pine Forests 21-28 Years Post-Mountain Pine Beetle Epidemic in Central Oregon
Source: PLoS One. 2014 Sep 15;9(9):e107532. doi: 10.1371/journal.pone.0107532 (PMC4164639; doi:10.1371/journal.pone.0107532)
Supplement: Table S5 — BIC table for the natural logarithm of canopy volume model. (DOCX) [file pone.0107532.s005.docx]

| **Model** | **df** | **BIC** | **ΔBIC** | **BIC weight** | **Evidence ratio** |
| --- | --- | --- | --- | --- | --- |
| ***Log(CV) = β_0_ + b_j_ + β_1_DMR_ij_ + ε_ij_*** | 4 | 59.11 | 0 | 7.95E-03 | 1 |
| ***Log(CV) = β_0_ + b_j_ + β_1_DMR_ij_ + β_2_PROD.L_ij_ + β_3_PROD.M_ij_ + ε_ij_*** | 6 | 59.53 | 0.41 | 6.47E-03 | 1.23 |
| ***Log(CV) = β_0_ + b_j_ + β_1_DMR_ij_ + β_2_SD_ij_ + β_3_PROD.L_ij_ + β_4_PROD.M_ij_ + ε_ij_*** | 7 | 60.67 | 1.56 | 3.65E-03 | 2.18 |
| ***Log(CV) = β_0_ + b_j_ + β_1_DMR_ij_ + β_2_SD_ij_ + ε_ij_*** | 5 | 62.30 | 3.19 | 1.61E-03 | 4.93 |
| ***Log(CV) = β_0_ + b_j_ + β_1_DMR_ij_ + β_2_SD_ij_ + β_3_DMR*SD_ij_ + ε_ij_*** | 6 | 62.45 | 3.34 | 1.50E-03 | 5.31 |
| ***Log(CV) = β_0_ + b_j_ + β_1_PROD.L_ij_ + β_2_PROD.M_ij_ + ε_ij_*** | 5 | 64.18 | 5.07 | 6.30E-04 | 12.62 |
| ***Log(CV) = β_0_ + b_j_ + β_1_DMR_ij_ + β_2_MPBMORT.L_ij_ + β_3_MPBMORT.M_ij_ + β_4_PROD.L_ij_ + β_5_PROD.L_ij_ + ε_ij_*** | 8 | 64.73 | 5.62 | 4.79E-04 | 16.59 |
| ***Log(CV) = β_0_ + b_j_ + β_1_SD_ij_ + ε_ij_*** | 4 | 64.83 | 5.71 | 4.57E-04 | 17.39 |
| ***Log(CV) = β_0_ + b_j_ + β_1_DMR_ij_ + β_2_MPBMORT.L_ij_ + β_3_MPBMORT.M_ij_ + β_4_PROD.L_ij_ + β_5_PROD.M_ij_ + β_6_SD_ij_ + ε_ij_*** | 9 | 65.24 | 6.12 | 3.72E-04 | 21.35 |
| ***Log(CV) = β_0_ + b_j_ + β_1_DMR_ij_ + β_2_PROD.L_ij_ + β_3_PROD.M_ij_ + β_4_DMR*PROD.L_ij_ + β_5_DMR*PROD.M_ij_ + ε_ij_*** | 8 | 65.59 | 6.48 | 3.12E-04 | 25.48 |
| ***Log(CV) = β_0_ + b_j_ + β_1_DMR_ij_ + β_2_MPBMORT.L_ij_ + β_3_MPBMORT.M_ij_ + ε_ij_*** | 6 | 65.71 | 6.60 | 2.93E-04 | 27.10 |
| ***Log(CV) = β_0_ + b_j_ + β_1_MPBMORT.L_ij_ + β_2_MPBMORT.M_ij_ + ε_ij_*** | 5 | 66.47 | 7.36 | 2.00E-04 | 39.64 |
| ***Log(CV) = β_0_ + b_j_ + β_1_DMR_ij_ + β_2_SD_ij_ + β_3_PROD.L_ij_ + β_4_PROD.M_ij_ + β_5_SD*DMR_ij_ + β_6_PROD.L*DMR_ij_ + β_7_PROD.M*DMR_ij_ + ε_ij_*** | 10 | 66.59 | 7.48 | 1.89E-04 | 42.11 |
| ***Log(CV) = β_0_ + b_j_ + β_1_DMR_ij_ + β_2_MPBMORT.L_ij_ + β_3_MPBMORT.M_ij_ + β_4_DMR*MPBMORT.L_ij_ + β_5_DMR*MPBMORT.M_ij_ + ε_ij_*** | 8 | 68.40 | 9.28 | 7.67E-05 | 103.65 |
| ***Log(CV) = β_0_ + b_j_ + β_1_DMR_ij_ + β_2_SD_ij_ + β_3_MPBMORT.L_ij_ + β_4_MPBMORT.M_ij_ + ε_ij_*** | 7 | 69.05 | 9.94 | 5.52E-05 | 143.87 |
| ***Log(CV) = β_0_ + b_j_ + β_1_DMR_ij_ + β_2_SD_ij_ + β_3_MPBMORT.L_ij_ + β_4_MPBMORT.M_ij_ + β_5_SD*DMR_ij_ + β_6_MPBMORT.L*DMR_ij_ + β_7_MPBMORT.M*DMR_ij_ + ε_ij_*** | 10 | 70.11 | 11.00 | 3.25E-05 | 244.79 |
| ***Log(CV) = β_0_ + b_j_ + β_1_DMR_ij_ + β_2_MPBMORT.L_ij_ + β_3_MPBMORT.M_ij_ + β_4_PROD.L_ij_ + β_5_PROD.M_ij_ + β_6_PROD.L*DMR_ij_ + β_7_PROD.M*DMR_ij_ +β_8_MPBMORT.L*DMR_ij_ + β_9_MPBMORT.M*DMR_ij_ + ε_ij_*** | 12 | 76.97 | 17.86 | 1.05E-06 | 7539.68 |
| ***Log(CV) = β_0_ + b_j_ + β_1_DMR_ij_ + β_2_MPBMORT.L_ij_ + β_3_MPBMORT.M_ij_ + β_4_PROD.L_ij_ + β_5_PROD.M_ij_ + β_6_SD_ij_ + β_7_PROD.L*DMR_ij_ + β_8_PROD.M*DMR_ij_ + β_9_MPBMORT.L*DMR_ij_ + β_10_MPBMORT.M*DMR_ij_ + β_11_SD*DMR_ij_ + ε_ij_*** | 14 | 77.92 | 18.81 | 6.55E-07 | 12138.95 |

**Table S5.** BIC table for the natural logarithm of canopy volume model.

Note: df= degrees of freedom; BIC = Bayesian Information Criterion; ΔBIC = difference in BIC value as compared with that of the preferred model; *Log(CV)_ij_* = natural logarithm of the canopy volume of the *ith* stand within the *jth* site; *β_0_* = mean of the natural logarithm of canopy volume when all additional *β’*s = 0; *SD_ij_* = stand density of the *ith* stand within the *jth* site; *DMR*_ij_ = dwarf mistletoe rating of the *ith* stand within the *jth* site; *PROD.L_ij_* = indicator which = 1 when the productivity of the *ith* stand within the *jth* site is low and 0 otherwise; *PROD.M_ij_* = indicator which = 1 when the productivity of the *ith* stand within the *jth* site is moderate and 0 otherwise; *MPBMORT.L_ij_* = indicator which = 1 when the mortality density of the previous mountain pine beetle epidemic of the *ith* stand within the *jth* site is low and 0 otherwise; *MPBMORT.L_ij_* = indicator which = 1 when the mortality density of the previous mountain pine beetle epidemic of the *ith* stand within the *jth* site is moderate and 0 otherwise; *b_j_* = random error for the *jth* site; *b_j_* ~ N(0, σ_b_^2^) and *b_j_* and *b_j’_* are independent; ***ε_ij_*** = random error from the natural logarithm of canopy volume measurements *ith* stand replicate within the *jth* site, ***ε_ij_*** ~ N(0, σ^2^) and ***ε_ij_*** and ***ε_i’j’_*** are independent.
