# Supplementary material for: Effects of Dwarf Mistletoe on Stand Structure of Lodgepole Pine Forests 21-28 Years Post-Mountain Pine Beetle Epidemic in Central Oregon
Source: PLoS One. 2014 Sep 15;9(9):e107532. doi: 10.1371/journal.pone.0107532 (PMC4164639; doi:10.1371/journal.pone.0107532)
Supplement: Table S6 — BIC table for the proportion of lodgepole pine in the dominant/codominant cohort model. (DOCX) [file pone.0107532.s006.docx]

**Table S6.** BIC table for the proportion of lodgepole pine in the dominant/codominant cohort model.

| **Model** | **df** | **BIC** | **ΔBIC** | **BIC weight** | **Evidence ratio** |
| --- | --- | --- | --- | --- | --- |
| ***logit(PD_ij_) = β_0_ + b_j_ + c_ij_ + β_1_DMR_ij_*** | 4 | 283.08 | 0 | 5.37E-03 | 1 |
| ***logit(PD_ij_) = β_0_ + b_j_ + c_ij_ + β_1_DMR_ij_ + β_2_SD_ij_*** | 5 | 286.12 | 3.04 | 1.17E-03 | 4.57 |
| ***logit(PD_ij_) = β_0_ + b_j_ + c_ij_ + β_1_DMR_ij_ + β_2_SD_ij_ + β_3_DMR*SD_ij_*** | 6 | 287.54 | 4.46 | 5.77E-04 | 9.30 |
| ***logit(PD_ij_) = β_0_ + b_j_ + c_ij_ + β_1_SD_ij_*** | 4 | 287.60 | 4.52 | 5.60E-04 | 9.58 |
| ***logit(PD_ij_) = β_0_ + b_j_ + c_ij_ + β_1_DMR_ij_ + β_2_PROD.L_ij_ + β_3_PROD.M_ij_*** | 6 | 288.31 | 5.22 | 3.95E-04 | 13.60 |
| ***logit(PD_ij_) = β_0_ + b_j_ + c_ij_ + β_1_DMR_ij_ + β_2_MPBMORT.L_ij_ + β_3_MPBMORT.M_ij_*** | 6 | 288.60 | 5.51 | 3.42E-04 | 15.72 |
| ***logit(PD_ij_) = β_0_ + b_j_ + c_ij_ + β_1_PROD.L_ij_ + β_2_PROD.M_ij_*** | 5 | 290.89 | 7.81 | 1.08E-04 | 49.65 |
| ***logit(PD_ij_) = β_0_ + b_j_ + c_ij_ + β_1_DMR_ij_ + β_2_SD_ij_ + β_3_PROD.L_ij_ + β_4_PROD.M_ij_*** | 7 | 291.66 | 8.57 | 7.40E-05 | 72.60 |
| ***logit(PD_ij_) = β_0_ + b_j_ + c_ij_ + β_1_DMR_ij_ + β_2_SD_ij_ + β_3_MPBMORT.L_ij_ + β_4_MPBMORT.M_ij_*** | 7 | 291.96 | 8.88 | 6.34E-05 | 84.77 |
| ***logit(PD_ij_) = β_0_ + b_j_ + c_ij_ + β_1_MPBMORT.L_ij_ + β_2_MPBMORT.M_ij_*** | 5 | 292.10 | 9.02 | 5.91E-05 | 90.92 |
| ***logit(PD_ij_) = β_0_ + b_j_ + c_ij_ + β_1_DMR_ij_ + β_2_PROD.L_ij_ + β_3_PROD.M_ij_ + β_4_DMR*PROD.L_ij_ + β_5_DMR*PROD.M_ij_*** | 8 | 292.91 | 9.83 | 3.94E-05 | 136.32 |
| ***logit(PD_ij_) = β_0_ + b_j_ + c_ij_ + β_1_DMR_ij_ + β_2_MPBMORT.L_ij_ + β_3_MPBMORT.M_ij_ + β_4_PROD.L_ij_ + β_5_PROD.L_ij_*** | 8 | 294.96 | 11.88 | 1.41E-05 | 379.93 |
| ***logit(PD_ij_) = β_0_ + b_j_ + c_ij_ + β_1_DMR_ij_ + β_2_MPBMORT.L_ij_ + β_3_MPBMORT.M_ij_ + β_4_DMR*MPBMORT.L_ij_ + β_5_DMR*MPBMORT.M_ij_*** | 8 | 295.27 | 12.19 | 1.21E-05 | 443.63 |
| ***logit(PD_ij_) = β_0_ + b_j_ + c_ij_ + β_1_DMR_ij_ + β_2_SD_ij_ + β_3_PROD.L_ij_ + β_4_PROD.M_ij_ + β_5_SD*DMR_ij_ + β_6_PROD.L*DMR_ij_ + β_7_PROD.M*DMR_ij_*** | 10 | 296.61 | 13.53 | 6.20E-06 | 866.97 |
| ***logit(PD_ij_) = β_0_ + b_j_ + c_ij_ + β_1_DMR_ij_ + β_2_MPBMORT.L_ij_ + β_3_MPBMORT.M_ij_ + β_4_PROD.L_ij_ + β_5_PROD.M_ij_ + β_6_SD_ij_*** | 9 | 298.42 | 15.34 | 2.51E-06 | 2143.08 |
| ***logit(PD_ij_) = β_0_ + b_j_ + c_ij_ + β_1_DMR_ij_ + β_2_SD_ij_ + β_3_MPBMORT.L_ij_ + β_4_MPBMORT.M_ij_ + β_5_SD*DMR_ij_ + β_6_MPBMORT.L*DMR_ij_ + β_7_MPBMORT.M*DMR_ij_*** | 10 | 300.32 | 17.24 | 9.69E-07 | 5541.39 |
| ***logit(PD_ij_) = β_0_ + b_j_ + c_ij_ + β_1_DMR_ij_ + β_2_MPBMORT.L_ij_ + β_3_MPBMORT.M_ij_ + β_4_PROD.L_ij_ + β_5_PROD.M_ij_ + β_6_PROD.L*DMR_ij_ + β_7_PROD.M*DMR_ij_ +β_8_MPBMORT.L*DMR_ij_ + β_9_MPBMORT.M*DMR_ij_*** | 12 | 305.48 | 22.40 | 7.34E-08 | 73130.44 |
| ***logit(PD_ij_) = β_0_ + b_j_ + c_ij_ + β_1_DMR_ij_ + β_2_MPBMORT.L_ij_ + β_3_MPBMORT.M_ij_ + β_4_PROD.L_ij_ + β_5_PROD.M_ij_ + β_6_SD_ij_ + β_7_PROD.L*DMR_ij_ + β_8_PROD.M*DMR_ij_ + β_9_MPBMORT.L*DMR_ij_ + β_10_MPBMORT.M*DMR_ij_ + β_11_SD*DMR_ij_*** | 14 | 309.83 | 26.75 | 8.34E-09 | 643707.69 |

Note: df= degrees of freedom; BIC = Bayesian Information Criterion; ΔBIC = difference in BIC value as compared with that of the preferred model; *logit(PD_ij_)* = the log odds that a lodgepole pine in the *ith* plot within the *jth* stand is in the dominant/codominant cohort; *β_0_* = the log odds that a lodgepole pine is in the dominant/codominant cohort when all additional *β’*s = 0; *SD_ij_* = stand density of the *ith* plot within the *jth* stand; *DMR*_ij_ = dwarf mistletoe rating of the *ith* plot within the *jth* stand; *PROD.L_ij_* = indicator which = 1 when the productivity of the *ith* plot within the *jth* stand is low and 0 otherwise; *PROD.M_ij_* = indicator which = 1 when the productivity of the *ith* plot within the *jth* stand is moderate and 0 otherwise; *MPBMORT.L_ij_* = indicator which = 1 when the mortality density of the previous mountain pine beetle epidemic of the *ith* plot within the *jth* stand is low and 0 otherwise; *MPBMORT.L_ij_* = indicator which = 1 when the mortality density of the previous mountain pine beetle epidemic of the *ith* plot within the *jth* stand is moderate and 0 otherwise; *b_j_* = random error for the *jth* stand; *b_j_* ~ B(n, p_b_) and *b_j_* and *b_j’_* are independent; *c_ij_* = random error from the *ith* plot replicate within the *jth* stand, *c_ij_* ~ B(n, p_c_) and *c_ij_* and *c_i’j’_* are independent.
