# Supplementary material for: Effects of Dwarf Mistletoe on Stand Structure of Lodgepole Pine Forests 21-28 Years Post-Mountain Pine Beetle Epidemic in Central Oregon
Source: PLoS One. 2014 Sep 15;9(9):e107532. doi: 10.1371/journal.pone.0107532 (PMC4164639; doi:10.1371/journal.pone.0107532)
Supplement: Table S7 — BIC table for the proportion of lodgepole pine in the suppressed cohort model. (DOCX) [file pone.0107532.s007.docx]

**Table S7.** BIC table for the proportion of lodgepole pine in the suppressed cohort model.

| **Model** | **df** | **BIC** | **ΔBIC** | **BIC weight** | **Evidence ratio** |
| --- | --- | --- | --- | --- | --- |
| ***logit(PS_ij_) = β_0_ + b_j_ + c_ij_ + β_1_DMR_ij_*** | 4 | 290.06 | 0 | 4.99E-03 | 1.00 |
| ***logit(PS_ij_) = β_0_ + b_j_ + c_ij_ + β_1_DMR_ij_ + β_2_SD_ij_*** | 5 | 293.70 | 3.64 | 8.09E-04 | 6.17 |
| ***logit(PS_ij_) = β_0_ + b_j_ + c_ij_ + β_1_DMR_ij_ + β_2_PROD.L_ij_ + β_3_PROD.M_ij_*** | 6 | 293.91 | 3.86 | 7.25E-04 | 6.89 |
| ***logit(PS_ij_) = β_0_ + b_j_ + c_ij_ + β_1_DMR_ij_ + β_2_MPBMORT.L_ij_ + β_3_MPBMORT.M_ij_*** | 6 | 296.18 | 6.12 | 2.34E-04 | 21.33 |
| ***logit(PS_ij_) = β_0_ + b_j_ + c_ij_ + β_1_DMR_ij_ + β_2_SD_ij_ + β_3_DMR*SD_ij_*** | 6 | 297.09 | 7.03 | 1.49E-04 | 33.62 |
| ***logit(PS_ij_) = β_0_ + b_j_ + c_ij_ + β_1_DMR_ij_ + β_2_SD_ij_ + β_3_PROD.L_ij_ + β_4_PROD.M_ij_*** | 7 | 297.52 | 7.47 | 1.19E-04 | 41.89 |
| ***logit(PS_ij_) = β_0_ + b_j_ + c_ij_ + β_1_DMR_ij_ + β_2_PROD.L_ij_ + β_3_PROD.M_ij_ + β_4_DMR*PROD.L_ij_ + β_5_DMR*PROD.M_ij_*** | 8 | 298.02 | 7.96 | 9.33E-05 | 53.52 |
| ***logit(PS_ij_) = β_0_ + b_j_ + c_ij_ + β_1_SD_ij_*** | 4 | 298.83 | 8.77 | 6.22E-05 | 80.24 |
| ***logit(PS_ij_) = β_0_ + b_j_ + c_ij_ + β_1_DMR_ij_ + β_2_SD_ij_ + β_3_MPBMORT.L_ij_ + β_4_MPBMORT.M_ij_*** | 7 | 299.83 | 9.77 | 3.78E-05 | 132.29 |
| ***logit(PS_ij_) = β_0_ + b_j_ + c_ij_ + β_1_PROD.L_ij_ + β_2_PROD.M_ij_*** | 5 | 300.53 | 10.48 | 2.65E-05 | 188.67 |
| ***logit(PS_ij_) = β_0_ + b_j_ + c_ij_ + β_1_DMR_ij_ + β_2_MPBMORT.L_ij_ + β_3_MPBMORT.M_ij_ + β_4_PROD.L_ij_ + β_5_PROD.L_ij_*** | 8 | 300.94 | 10.89 | 2.16E-05 | 231.60 |
| ***logit(PS_ij_) = β_0_ + b_j_ + c_ij_ + β_1_MPBMORT.L_ij_ + β_2_MPBMORT.M_ij_*** | 5 | 301.93 | 11.87 | 1.32E-05 | 378.04 |
| ***logit(PS_ij_) = β_0_ + b_j_ + c_ij_ + β_1_DMR_ij_ + β_2_MPBMORT.L_ij_ + β_3_MPBMORT.M_ij_ + β_4_DMR*MPBMORT.L_ij_ + β_5_DMR*MPBMORT.M_ij_*** | 8 | 303.34 | 13.28 | 6.53E-06 | 765.09 |
| ***logit(PS_ij_) = β_0_ + b_j_ + c_ij_ + β_1_DMR_ij_ + β_2_SD_ij_ + β_3_PROD.L_ij_ + β_4_PROD.M_ij_ + β_5_SD*DMR_ij_ + β_6_PROD.L*DMR_ij_ + β_7_PROD.M*DMR_ij_*** | 10 | 304.38 | 14.32 | 3.88E-06 | 1286.91 |
| ***logit(PS_ij_) = β_0_ + b_j_ + c_ij_ + β_1_DMR_ij_ + β_2_MPBMORT.L_ij_ + β_3_MPBMORT.M_ij_ + β_4_PROD.L_ij_ + β_5_PROD.M_ij_ + β_6_SD_ij_*** | 9 | 304.48 | 14.42 | 3.69E-06 | 1352.89 |
| ***logit(PS_ij_) = β_0_ + b_j_ + c_ij_ + β_1_DMR_ij_ + β_2_SD_ij_ + β_3_MPBMORT.L_ij_ + β_4_MPBMORT.M_ij_ + β_5_SD*DMR_ij_ + β_6_MPBMORT.L*DMR_ij_ + β_7_MPBMORT.M*DMR_ij_*** | 10 | 310.32 | 20.26 | 1.99E-07 | 25084.36 |
| ***logit(PS_ij_) = β_0_ + b_j_ + c_ij_ + β_1_DMR_ij_ + β_2_MPBMORT.L_ij_ + β_3_MPBMORT.M_ij_ + β_4_PROD.L_ij_ + β_5_PROD.M_ij_ + β_6_PROD.L*DMR_ij_ + β_7_PROD.M*DMR_ij_ +β_8_MPBMORT.L*DMR_ij_ + β_9_MPBMORT.M*DMR_ij_*** | 12 | 311.81 | 21.75 | 9.45E-08 | 52838.74 |
| ***logit(PS_ij_) = β_0_ + b_j_ + c_ij_ + β_1_DMR_ij_ + β_2_MPBMORT.L_ij_ + β_3_MPBMORT.M_ij_ + β_4_PROD.L_ij_ + β_5_PROD.M_ij_ + β_6_SD_ij_ + β_7_PROD.L*DMR_ij_ + β_8_PROD.M*DMR_ij_ + β_9_MPBMORT.L*DMR_ij_ + β_10_MPBMORT.M*DMR_ij_ + β_11_SD*DMR_ij_*** | 14 | 318.38 | 28.32 | 3.54E-09 | 1411269.20 |

Note: df= degrees of freedom; BIC = Bayesian Information Criterion; ΔBIC = difference in BIC value as compared with that of the preferred model; *logit(PS_ij_)* = the log odds that a lodgepole pine in the *ith* plot within the *jth* stand is in the suppressed cohort; *β_0_* = the log odds that a lodgepole pine is in the suppressed cohort when all additional *β’*s = 0; *SD_ij_* = stand density of the *ith* plot within the *jth* stand; *DMR*_ij_ = dwarf mistletoe rating of the *ith* plot within the *jth* stand; *PROD.L_ij_* = indicator which = 1 when the productivity of the *ith* plot within the *jth* stand is low and 0 otherwise; *PROD.M_ij_* = indicator which = 1 when the productivity of the *ith* plot within the *jth* stand is moderate and 0 otherwise; *MPBMORT.L_ij_* = indicator which = 1 when the mortality density of the previous mountain pine beetle epidemic of the *ith* plot within the *jth* stand is low and 0 otherwise; *MPBMORT.L_ij_* = indicator which = 1 when the mortality density of the previous mountain pine beetle epidemic of the *ith* plot within the *jth* stand is moderate and 0 otherwise; *b_j_* = random error for the *jth* stand; *b_j_* ~ B(n, p_b_) and *b_j_* and *b_j’_* are independent; *c_ij_* = random error from the *ith* plot replicate within the *jth* stand, *c_ij_* ~ B(n, p_c_) and *c_ij_* and *c_i’j’_* are independent.
