# Supplementary material for: Effects of Dwarf Mistletoe on Stand Structure of Lodgepole Pine Forests 21-28 Years Post-Mountain Pine Beetle Epidemic in Central Oregon
Source: PLoS One. 2014 Sep 15;9(9):e107532. doi: 10.1371/journal.pone.0107532 (PMC4164639; doi:10.1371/journal.pone.0107532)
Supplement: Table S8 — BIC table for the proportion of lodgepole pine in the intermediate cohort model. (DOCX) [file pone.0107532.s008.docx]

**Table S8.** BIC table for the proportion of lodgepole pine in the intermediate cohort model.

| **Model** | **df** | **BIC** | **ΔBIC** | **BIC weight** | **Evidence ratio** |
| --- | --- | --- | --- | --- | --- |
| ***logit(PI_i_) = β_0_ + b_i_ + β_1_SD_i_*** | 3 | 238.02 | 0 | 4.87E-03 | 1.00 |
| ***logit(PI_i_) = β_0_ + b_i_ + β_1_DMR_i_*** | 3 | 240.16 | 2.10 | 1.70E-03 | 2.86 |
| ***logit(PI_i_) = β_0_ + b_i_ + β_1_DMR_i_ + β_2_SD_i_*** | 4 | 241.04 | 3.02 | 1.08E-03 | 4.53 |
| ***logit(PI_i_) = β_0_ + b_i_ + β_1_MPBMORT.L_i_ + β_2_MPBMORT.M_i_*** | 4 | 241.14 | 3.12 | 1.02E-03 | 4.76 |
| ***logit(PI_i_) = β_0_ + b_i_ + β_1_PROD.L_i_ + β_2_PROD.M_i_*** | 4 | 243.38 | 5.36 | 3.34E-04 | 14.59 |
| ***logit(PI_i_) = β_0_ + b_i_ + β_1_DMR_i_ + β_2_SD_i_ + β_3_DMR*SD_i_*** | 5 | 243.56 | 5.54 | 3.05E-04 | 15.96 |
| ***logit(PI_i_) = β_0_ + b_i_ + β_1_DMR_i_ + β_2_MPBMORT.L_i_ + β_3_MPBMORT.M_i_*** | 5 | 244.80 | 6.78 | 1.64E-04 | 29.67 |
| ***logit(PI_i_) = β_0_ + b_i_ + β_1_DMR_i_ + β_2_SD_i_ + β_3_MPBMORT.L_i_ + β_4_MPBMORT.M_i_*** | 6 | 246.39 | 8.37 | 7.42E-05 | 65.69 |
| ***logit(PI_i_) = β_0_ + b_i_ + β_1_DMR_i_ + β_2_PROD.L_i_ + β_3_PROD.M_i_*** | 5 | 246.63 | 8.62 | 6.54E-05 | 74.44 |
| ***logit(PI_i_) = β_0_ + b_i_ + β_1_DMR_i_ + β_2_SD_i_ + β_3_PROD.L_i_ + β_4_PROD.M_i_*** | 7 | 247.23 | 9.21 | 4.87E-05 | 99.98 |
| ***logit(PI_i_) = β_0_ + b_i_ + β_1_DMR_i_ + β_2_MPBMORT.L_i_ + β_3_MPBMORT.M_i_ + β_4_DMR*MPBMORT.L_i_ + β_5_DMR*MPBMORT.M_i_*** | 7 | 251.36 | 13.34 | 6.18E-06 | 788.40 |
| ***logit(PI_i_) = β_0_ + b_i_ + β_1_DMR_i_ + β_2_MPBMORT.L_i_ + β_3_MPBMORT.M_i_ + β_4_PROD.L_i_ + β_5_PROD.L_i_*** | 7 | 251.64 | 13.62 | 5.37E-06 | 906.87 |
| ***logit(PI_i_) = β_0_ + b_i_ + β_1_DMR_i_ + β_2_MPBMORT.L_i_ + β_3_MPBMORT.M_i_ + β_4_PROD.L_i_ + β_5_PROD.M_i_ + β_6_SD_i_*** | 8 | 252.90 | 14.88 | 2.86E-06 | 1702.75 |
| ***logit(PI_i_) = β_0_ + b_i_ + β_1_DMR_i_ + β_2_PROD.L_i_ + β_3_PROD.M_i_ + β_4_DMR*PROD.L_i_ + β_5_DMR*PROD.M_i_*** | 7 | 253.81 | 15.79 | 1.82E-06 | 2683.83 |
| ***logit(PI_i_) = β_0_ + b_i_ + β_1_DMR_i_ + β_2_SD_i_ + β_3_PROD.L_i_ + β_4_PROD.M_i_ + β_5_SD*DMR_i_ + β_6_PROD.L*DMR_i_ + β_7_PROD.M*DMR_i_*** | 9 | 256.65 | 18.63 | 4.39E-07 | 11103.33 |
| ***logit(PI_i_) = β_0_ + b_i_ + β_1_DMR_i_ + β_2_SD_i_ + β_3_MPBMORT.L_i_ + β_4_MPBMORT.M_i_ + β_5_SD*DMR_i_ + β_6_MPBMORT.L*DMR_i_ + β_7_MPBMORT.M*DMR_i_*** | 9 | 256.66 | 18.64 | 4.37E-07 | 11158.98 |
| ***logit(PI_i_) = β_0_ + b_i_ + β_1_DMR_i_ + β_2_MPBMORT.L_i_ + β_3_MPBMORT.M_i_ + β_4_PROD.L_i_ + β_5_PROD.M_i_ + β_6_PROD.L*DMR_i_ + β_7_PROD.M*DMR_i_ +β_8_MPBMORT.L*DMR_i_ + β_9_MPBMORT.M*DMR_i_*** | 11 | 264.83 | 26.81 | 7.34E-09 | 663311.50 |
| ***logit(PI_i_) = β_0_ + b_i_ + β_1_DMR_i_ + β_2_MPBMORT.L_i_ + β_3_MPBMORT.M_i_ + β_4_PROD.L_i_ + β_5_PROD.M_i_ + β_6_SD_i_ + β_7_PROD.L*DMR_i_ + β_8_PROD.M*DMR_i_ + β_9_MPBMORT.L*DMR_i_ + β_10_MPBMORT.M*DMR_i_ + β_11_SD*DMR_i_*** | 13 | 269.46 | 31.45 | 7.22E-10 | 6749641.84 |

Note: df= degrees of freedom; BIC = Bayesian Information Criterion; ΔBIC = difference in BIC value as compared with that of the preferred model; *logit(PI_i_)* = the log odds that a lodgepole pine in a plot within the *ith* stand is in the intermediate cohort; *β_0_* = the log odds that a lodgepole pine is in the intermediate cohort when all additional *β’*s = 0; *SD_i_* = stand density of a plot within the *ith* stand; *DMR*_i_ = dwarf mistletoe rating of a plot within the *ith* stand; *PROD.L_i_* = indicator which = 1 when the productivity of a plot within the *ith* stand is low and 0 otherwise; *PROD.M_i_* = indicator which = 1 when the productivity of a plot within the *ith* stand is moderate and 0 otherwise; *MPBMORT.L_i_* = indicator which = 1 when the mortality density of the previous mountain pine beetle epidemic of a plot within the *ith* stand is low and 0 otherwise; *MPBMORT.L_i_* = indicator which = 1 when the mortality density of the previous mountain pine beetle epidemic of a plot within the *ith* stand is moderate and 0 otherwise; *b_j_* = random error for the *ith* stand; *b_i_* ~ B(n, p_b_) and *b_i_* and *b_i’_* are independent.
