# Supplementary material for: Effects of Dwarf Mistletoe on Stand Structure of Lodgepole Pine Forests 21-28 Years Post-Mountain Pine Beetle Epidemic in Central Oregon
Source: PLoS One. 2014 Sep 15;9(9):e107532. doi: 10.1371/journal.pone.0107532 (PMC4164639; doi:10.1371/journal.pone.0107532)
Supplement: Table S9 — BIC table for the cohort height of intermediates model. (DOCX) [file pone.0107532.s009.docx]

**Table S9.** BIC table for the cohort height of intermediates model.

| **Model** | **df** | **BIC** | **ΔBIC** | **BIC weight** | **Evidence ratio** |
| --- | --- | --- | --- | --- | --- |
| ***CHI_ij_ = β_0_ + b_j_ + β_1_DMR_ij_ + ε_ij_*** | 4 | 179.23 | 0 | 5.20E-03 | 1 |
| ***CHI_ij_ = β_0_ + b_j_ + β_1_SD_ij_ + ε_ij_*** | 4 | 182.22 | 2.99 | 1.17E-03 | 4.45 |
| ***CHI_ij_ = β_0_ + b_j_ + β_1_DMR_ij_ + β_2_SD_ij_ + ε_ij_*** | 5 | 182.29 | 3.06 | 1.13E-03 | 4.61 |
| ***CHI_ij_ = β_0_ + b_j_ + β_1_DMR_ij_ + β_2_MPBMORT.L_ij_ + β_3_MPBMORT.M_ij_ + ε_ij_*** | 6 | 184.46 | 5.22 | 3.82E-04 | 13.62 |
| ***CHI_ij_ = β_0_ + b_j_ + β_1_DMR_ij_ + β_2_PROD.L_ij_ + β_3_PROD.M_ij_ + ε_ij_*** | 6 | 184.65 | 5.41 | 3.47E-04 | 14.98 |
| ***CHI_ij_ = β_0_ + b_j_ + β_1_DMR_ij_ + β_2_SD_ij_ + β_3_DMR*SD_ij_ + ε_ij_*** | 6 | 185.03 | 5.80 | 2.86E-04 | 18.16 |
| ***CHI_ij_ = β_0_ + b_j_ + β_1_PROD.L_ij_ + β_2_PROD.M_ij_ + ε_ij_*** | 5 | 185.07 | 5.83 | 2.81E-04 | 18.49 |
| ***CHI_ij_ = β_0_ + b_j_ + β_1_MPBMORT.L_ij_ + β_2_MPBMORT.M_ij_ + ε_ij_*** | 5 | 185.73 | 6.50 | 2.02E-04 | 25.76 |
| ***CHI_ij_ = β_0_ + b_j_ + β_1_DMR_ij_ + β_2_SD_ij_ + β_3_MPBMORT.L_ij_ + β_4_MPBMORT.M_ij_ + ε_ij_*** | 7 | 187.74 | 8.50 | 7.41E-05 | 70.24 |
| ***CHI_ij_ = β_0_ + b_j_ + β_1_DMR_ij_ + β_2_SD_ij_ + β_3_PROD.L_ij_ + β_4_PROD.M_ij_ + ε_ij_*** | 7 | 188.00 | 8.77 | 6.49E-05 | 80.14 |
| ***CHI_ij_ = β_0_ + b_j_ + β_1_DMR_ij_ + β_2_MPBMORT.L_ij_ + β_3_MPBMORT.M_ij_ + β_4_DMR*MPBMORT.L_ij_ + β_5_DMR*MPBMORT.M_ij_ + ε_ij_*** | 8 | 190.38 | 11.15 | 1.97E-05 | 263.49 |
| ***CHI_ij_ = β_0_ + b_j_ + β_1_DMR_ij_ + β_2_MPBMORT.L_ij_ + β_3_MPBMORT.M_ij_ + β_4_PROD.L_ij_ + β_5_PROD.L_ij_ + ε_ij_*** | 8 | 190.79 | 11.55 | 1.61E-05 | 322.51 |
| ***CHI_ij_ = β_0_ + b_j_ + β_1_DMR_ij_ + β_2_PROD.L_ij_ + β_3_PROD.M_ij_ + β_4_DMR*PROD.L_ij_ + β_5_DMR*PROD.M_ij_ + ε_ij_*** | 8 | 191.69 | 12.46 | 1.03E-05 | 507.53 |
| ***CHI_ij_ = β_0_ + b_j_ + β_1_DMR_ij_ + β_2_MPBMORT.L_ij_ + β_3_MPBMORT.M_ij_ + β_4_PROD.L_ij_ + β_5_PROD.M_ij_ + β_6_SD_ij_ + ε_ij_*** | 9 | 194.27 | 15.04 | 2.83E-06 | 1840.98 |
| ***CHI_ij_ = β_0_ + b_j_ + β_1_DMR_ij_ + β_2_SD_ij_ + β_3_MPBMORT.L_ij_ + β_4_MPBMORT.M_ij_ + β_5_SD*DMR_ij_ + β_6_MPBMORT.L*DMR_ij_ + β_7_MPBMORT.M*DMR_ij_ + ε_ij_*** | 10 | 196.11 | 16.87 | 1.13E-06 | 4613.57 |
| ***CHI_ij_ = β_0_ + b_j_ + β_1_DMR_ij_ + β_2_SD_ij_ + β_3_PROD.L_ij_ + β_4_PROD.M_ij_ + β_5_SD*DMR_ij_ + β_6_PROD.L*DMR_ij_ + β_7_PROD.M*DMR_ij_ + ε_ij_*** | 10 | 197.53 | 18.29 | 5.54E-07 | 9390.89 |
| ***CHI_ij_ = β_0_ + b_j_ + β_1_DMR_ij_ + β_2_MPBMORT.L_ij_ + β_3_MPBMORT.M_ij_ + β_4_PROD.L_ij_ + β_5_PROD.M_ij_ + β_6_PROD.L*DMR_ij_ + β_7_PROD.M*DMR_ij_ +β_8_MPBMORT.L*DMR_ij_ + β_9_MPBMORT.M*DMR_ij_ + ε_ij_*** | 12 | 203.78 | 24.55 | 2.43E-08 | 214280.4 |
| ***CHI_ij_ = β_0_ + b_j_ + β_1_DMR_ij_ + β_2_MPBMORT.L_ij_ + β_3_MPBMORT.M_ij_ + β_4_PROD.L_ij_ + β_5_PROD.M_ij_ + β_6_SD_ij_ + β_7_PROD.L*DMR_ij_ + β_8_PROD.M*DMR_ij_ + β_9_MPBMORT.L*DMR_ij_ + β_10_MPBMORT.M*DMR_ij_ + β_11_SD*DMR_ij_ + ε_ij_*** | 14 | 209.45 | 30.22 | 1.43E-09 | 3641783 |

Note: df= degrees of freedom; BIC = Bayesian Information Criterion; ΔBIC = difference in BIC value as compared with that of the preferred model; *CHI_ij_* = cohort height of intermediates of the *ith* stand within the *jth* site; *β_0_* = mean of the cohort height of intermediates when all additional *β’*s = 0; *SD_ij_* = stand density of the *ith* stand within the *jth* site; *DMR*_ij_ = dwarf mistletoe rating of the *ith* stand within the *jth* site; *PROD.L_ij_* = indicator which = 1 when the productivity of the *ith* stand within the *jth* site is low and 0 otherwise; *PROD.M_ij_* = indicator which = 1 when the productivity of the *ith* stand within the *jth* site is moderate and 0 otherwise; *MPBMORT.L_ij_* = indicator which = 1 when the mortality density of the previous mountain pine beetle epidemic of the *ith* stand within the *jth* site is low and 0 otherwise; *MPBMORT.L_ij_* = indicator which = 1 when the mortality density of the previous mountain pine beetle epidemic of the *ith* stand within the *jth* site is moderate and 0 otherwise; *b_j_* = random error for the *jth* site; *b_j_* ~ N(0, σ_b_^2^) and *b_j_* and *b_j’_* are independent; ***ε_ij_*** = random error from the cohort height of intermediates measurements *ith* stand replicate within the *jth* site, ***ε_ij_*** ~ N(0, σ^2^) and ***ε_ij_*** and ***ε_i’j’_*** are independent.
