# Supplementary material for: Effects of Dwarf Mistletoe on Stand Structure of Lodgepole Pine Forests 21-28 Years Post-Mountain Pine Beetle Epidemic in Central Oregon
Source: PLoS One. 2014 Sep 15;9(9):e107532. doi: 10.1371/journal.pone.0107532 (PMC4164639; doi:10.1371/journal.pone.0107532)
Supplement: Table S10 — BIC table for the cohort height of suppressed model. (DOCX) [file pone.0107532.s010.docx]

**Table S10.** BIC table for the cohort height of suppressed model.

| **Model** | **df** | **BIC** | **ΔBIC** | **BIC weight** | **Evidence ratio** |
| --- | --- | --- | --- | --- | --- |
| ***CHS_ij_ = β_0_ + b_j_ + β_1_DMR_ij_ + ε_ij_*** | 4 | 113.39 | 0 | 5.06E-03 | 1 |
| ***CHS_ij_ = β_0_ + b_j_ + β_1_DMR_ij_ + β_2_SD_ij_ + ε_ij_*** | 5 | 116.45 | 3.05 | 1.10E-03 | 4.61 |
| ***CHS_ij_ = β_0_ + b_j_ + β_1_DMR_ij_ + β_2_PROD.L_ij_ + β_3_PROD.M_ij_ + ε_ij_*** | 6 | 117.67 | 4.28 | 5.97E-04 | 8.48 |
| ***CHS_ij_ = β_0_ + b_j_ + β_1_DMR_ij_ + β_2_MPBMORT.L_ij_ + β_3_MPBMORT.M_ij_ + ε_ij_*** | 6 | 119.32 | 5.93 | 2.62E-04 | 19.36 |
| ***CHS_ij_ = β_0_ + b_j_ + β_1_DMR_ij_ + β_2_SD_ij_ + β_3_DMR*SD_ij_ + ε_ij_*** | 6 | 119.72 | 6.32 | 2.14E-04 | 23.61 |
| ***CHS_ij_ = β_0_ + b_j_ + β_1_DMR_ij_ + β_2_SD_ij_ + β_3_PROD.L_ij_ + β_4_PROD.M_ij_ + ε_ij_*** | 7 | 120.12 | 6.73 | 1.75E-04 | 28.92 |
| ***CHS_ij_ = β_0_ + b_j_ + β_1_SD_ij_ + ε_ij_*** | 4 | 120.29 | 6.90 | 1.61E-04 | 31.44 |
| ***CHS_ij_ = β_0_ + b_j_ + β_1_MPBMORT.L_ij_ + β_2_MPBMORT.M_ij_ + ε_ij_*** | 5 | 120.72 | 7.33 | 1.30E-04 | 39.05 |
| ***CHS_ij_ = β_0_ + b_j_ + β_1_PROD.L_ij_ + β_2_PROD.M_ij_ + ε_ij_*** | 5 | 122.12 | 8.73 | 6.45E-05 | 78.49 |
| ***CHS_ij_ = β_0_ + b_j_ + β_1_DMR_ij_ + β_2_SD_ij_ + β_3_MPBMORT.L_ij_ + β_4_MPBMORT.M_ij_ + ε_ij_*** | 7 | 122.64 | 9.24 | 4.98E-05 | 101.62 |
| ***CHS_ij_ = β_0_ + b_j_ + β_1_DMR_ij_ + β_2_MPBMORT.L_ij_ + β_3_MPBMORT.M_ij_ + β_4_PROD.L_ij_ + β_5_PROD.L_ij_ + ε_ij_*** | 8 | 124.39 | 10.99 | 2.08E-05 | 243.98 |
| ***CHS_ij_ = β_0_ + b_j_ + β_1_DMR_ij_ + β_2_MPBMORT.L_ij_ + β_3_MPBMORT.M_ij_ + β_4_DMR*MPBMORT.L_ij_ + β_5_DMR*MPBMORT.M_ij_ + ε_ij_*** | 8 | 125.58 | 12.18 | 1.14E-05 | 442.30 |
| ***CHS_ij_ = β_0_ + b_j_ + β_1_DMR_ij_ + β_2_MPBMORT.L_ij_ + β_3_MPBMORT.M_ij_ + β_4_PROD.L_ij_ + β_5_PROD.M_ij_ + β_6_SD_ij_ + ε_ij_*** | 9 | 127.08 | 13.68 | 5.41E-06 | 936.14 |
| ***CHS_ij_ = β_0_ + b_j_ + β_1_DMR_ij_ + β_2_SD_ij_ + β_3_PROD.L_ij_ + β_4_PROD.M_ij_ + β_5_SD*DMR_ij_ + β_6_PROD.L*DMR_ij_ + β_7_PROD.M*DMR_ij_ + ε_ij_*** | 10 | 129.93 | 16.53 | 1.30E-06 | 3891.92 |
| ***CHS_ij_ = β_0_ + b_j_ + β_1_DMR_ij_ + β_2_SD_ij_ + β_3_MPBMORT.L_ij_ + β_4_MPBMORT.M_ij_ + β_5_SD*DMR_ij_ + β_6_MPBMORT.L*DMR_ij_ + β_7_MPBMORT.M*DMR_ij_ + ε_ij_*** | 10 | 132.50 | 19.11 | 3.59E-07 | 14097.14 |
| ***CHS_ij_ = β_0_ + b_j_ + β_1_DMR_ij_ + β_2_MPBMORT.L_ij_ + β_3_MPBMORT.M_ij_ + β_4_PROD.L_ij_ + β_5_PROD.M_ij_ + β_6_PROD.L*DMR_ij_ + β_7_PROD.M*DMR_ij_ +β_8_MPBMORT.L*DMR_ij_ + β_9_MPBMORT.M*DMR_ij_ + ε_ij_*** | 12 | 138.07 | 24.67 | 2.22E-08 | 2.28E+05 |
| ***Y_ij_ = β_0_ + b_j_ + β_1_DMR_ij_ + β_2_MPBMORT.L_ij_ + β_3_MPBMORT.M_ij_ + β_4_PROD.L_ij_ + β_5_PROD.M_ij_ + β_6_SD_ij_ + β_7_PROD.L*DMR_ij_ + β_8_PROD.M*DMR_ij_ + β_9_MPBMORT.L*DMR_ij_ + β_10_MPBMORT.M*DMR_ij_ + β_11_SD*DMR_ij_ + ε_ij_*** | 14 | 144.39 | 31.00 | 9.40E-10 | 5.38E+06 |

Note: df= degrees of freedom; BIC = Bayesian Information Criterion; ΔBIC = difference in BIC value as compared with that of the preferred model; *CHS_ij_* = cohort height of suppressed of the *ith* stand within the *jth* site; *β_0_* = mean of the cohort height of suppressed when all additional *β’*s = 0; *SD_ij_* = stand density of the *ith* stand within the *jth* site; *DMR*_ij_ = dwarf mistletoe rating of the *ith* stand within the *jth* site; *PROD.L_ij_* = indicator which = 1 when the productivity of the *ith* stand within the *jth* site is low and 0 otherwise; *PROD.M_ij_* = indicator which = 1 when the productivity of the *ith* stand within the *jth* site is moderate and 0 otherwise; *MPBMORT.L_ij_* = indicator which = 1 when the mortality density of the previous mountain pine beetle epidemic of the *ith* stand within the *jth* site is low and 0 otherwise; *MPBMORT.L_ij_* = indicator which = 1 when the mortality density of the previous mountain pine beetle epidemic of the *ith* stand within the *jth* site is moderate and 0 otherwise; *b_j_* = random error for the *jth* site; *b_j_* ~ N(0, σ_b_^2^) and *b_j_* and *b_j’_* are independent; ***ε_ij_*** = random error from the cohort height of suppressed measurements *ith* stand replicate within the *jth* site, ***ε_ij_*** ~ N(0, σ^2^) and ***ε_ij_*** and ***ε_i’j’_*** are independent.
