# Supplementary material for: Effects of Dwarf Mistletoe on Stand Structure of Lodgepole Pine Forests 21-28 Years Post-Mountain Pine Beetle Epidemic in Central Oregon
Source: PLoS One. 2014 Sep 15;9(9):e107532. doi: 10.1371/journal.pone.0107532 (PMC4164639; doi:10.1371/journal.pone.0107532)
Supplement: Table S11 — BIC table for the cohort height of dominant/codominants model. (DOCX) [file pone.0107532.s011.docx]

**Table S11.** BIC table for the cohort height of dominant/codominants model.

| **Model** | **df** | **BIC** | **ΔBIC** | **BIC weight** | **Evidence ratio** |
| --- | --- | --- | --- | --- | --- |
| ***CHD_ij_ = β_0_ + b_j_ + β_1_SD_ij_ + ε_ij_*** | 4 | 173.99 | 0 | 4.67E-03 | 1 |
| ***CHD_ij_ = β_0_ + b_j_ + β_1_DMR_ij_ + β_2_SD_ij_ + ε_ij_*** | 5 | 175.34 | 1.36 | 2.37E-03 | 1.97 |
| ***CHD_ij_ = β_0_ + b_j_ + β_1_DMR_ij_ + β_2_SD_ij_ + β_3_DMR*SD_ij_ + ε_ij_*** | 6 | 177.69 | 3.71 | 7.32E-04 | 6.38 |
| ***CHD_ij_ = β_0_ + b_j_ + β_1_DMR_ij_ + β_2_SD_ij_ + β_3_PROD.L_ij_ + β_4_PROD.M_ij_ + ε_ij_*** | 7 | 177.86 | 3.88 | 6.72E-04 | 6.95 |
| ***CHD_ij_ = β_0_ + b_j_ + β_1_DMR_ij_ + ε_i_*** | 4 | 181.24 | 7.25 | 1.24E-04 | 37.57 |
| ***CHD_ij_ = β_0_ + b_j_ + β_1_DMR_ij_ + β_2_MPBMORT.L_ij_ + β_3_MPBMORT.M_ij_ + β_4_PROD.L_ij_ + β_5_PROD.M_ij_ + β_6_SD_ij_ + ε_ij_*** | 9 | 181.35 | 7.36 | 1.18E-04 | 39.71 |
| ***CHD_ij_ = β_0_ + b_j_ + β_1_DMR_ij_ + β_2_SD_ij_ + β_3_MPBMORT.L_ij_ + β_4_MPBMORT.M_ij_ + ε_ij_*** | 7 | 181.60 | 7.61 | 1.04E-04 | 44.96 |
| ***CHD_ij_ = β_0_ + b_j_ + β_1_DMR_ij_ + β_2_SD_ij_ + β_3_PROD.L_ij_ + β_4_PROD.M_ij_ + β_5_SD*DMR_ij_ + β_6_PROD.L*DMR_ij_ + β_7_PROD.M*DMR_ij_ + ε_ij_*** | 10 | 185.61 | 11.62 | 1.40E-05 | 334.39 |
| ***CHD_ij_ = β_0_ + b_j_ + β_1_DMR_ij_ + β_2_MPBMORT.L_ij_ + β_3_MPBMORT.M_ij_ + ε_ij_*** | 6 | 186.46 | 12.48 | 9.13E-06 | 511.69 |
| ***CHD_ij_ = β_0_ + b_j_ + β_1_DMR_ij_ + β_2_PROD.L_ij_ + β_3_PROD.M_ij_ + ε_ij_*** | 6 | 187.39 | 13.40 | 5.74E-06 | 813.92 |
| ***CHD_ij_ = β_0_ + b_j_ + β_1_PROD.L_ij_ + β_2_PROD.M_ij_ + ε_ij_*** | 5 | 187.48 | 13.49 | 5.50E-06 | 849.34 |
| ***CHD_ij_ = β_0_ + b_j_ + β_1_MPBMORT.L_ij_ + β_2_MPBMORT.M_ij_ + ε_ij_*** | 5 | 187.90 | 13.92 | 4.44E-06 | 1052.47 |
| ***CHD_ij_ = β_0_ + b_j_ + β_1_DMR_ij_ + β_2_SD_ij_ + β_3_MPBMORT.L_ij_ + β_4_MPBMORT.M_ij_ + β_5_SD*DMR_ij_ + β_6_MPBMORT.L*DMR_ij_ + β_7_MPBMORT.M*DMR_ij_ + ε_ij_*** | 10 | 189.66 | 15.68 | 1.84E-06 | 2534.19 |
| ***CHD_ij_ = β_0_ + b_j_ + β_1_DMR_ij_ + β_2_MPBMORT.L_ij_ + β_3_MPBMORT.M_ij_ + β_4_PROD.L_ij_ + β_5_PROD.L_ij_ + ε_ij_*** | 8 | 191.46 | 17.47 | 7.52E-07 | 6215.49 |
| ***CHD_ij_ = β_0_ + b_j_ + β_1_DMR_ij_ + β_2_MPBMORT.L_ij_ + β_3_MPBMORT.M_ij_ + β_4_DMR*MPBMORT.L_ij_ + β_5_DMR*MPBMORT.M_ij_ + ε_ij_*** | 8 | 192.38 | 18.39 | 4.74E-07 | 9853.35 |
| ***CHD_ij_ = β_0_ + b_j_ + β_1_DMR_ij_ + β_2_MPBMORT.L_ij_ + β_3_MPBMORT.M_ij_ + β_4_PROD.L_ij_ + β_5_PROD.M_ij_ + β_6_SD_ij_ + β_7_PROD.L*DMR_ij_ + β_8_PROD.M*DMR_ij_ + β_9_MPBMORT.L*DMR_ij_ + β_10_MPBMORT.M*DMR_ij_ + β_11_SD*DMR_ij_ + ε_ij_*** | 14 | 192.47 | 18.48 | 4.53E-07 | 10306.37 |
| ***CHD_ij_ = β_0_ + b_j_ + β_1_DMR_ij_ + β_2_PROD.L_ij_ + β_3_PROD.M_ij_ + β_4_DMR*PROD.L_ij_ + β_5_DMR*PROD.M_ij_ + ε_ij_*** | 8 | 193.66 | 19.67 | 2.50E-07 | 18675.16 |
| ***CHD_ij_ = β_0_ + b_j_ + β_1_DMR_ij_ + β_2_MPBMORT.L_ij_ + β_3_MPBMORT.M_ij_ + β_4_PROD.L_ij_ + β_5_PROD.M_ij_ + β_6_PROD.L*DMR_ij_ + β_7_PROD.M*DMR_ij_ + β_8_MPBMORT.L*DMR_ij_ + β_9_MPBMORT.M*DMR_ij_ + ε_ij_*** | 12 | 202.29 | 28.31 | 3.33E-09 | 1.40E+06 |

Note: df= degrees of freedom; BIC = Bayesian Information Criterion; ΔBIC = difference in BIC value as compared with that of the preferred model; *CHD_ij_* = cohort height of dominant/codominants of the *ith* stand within the *jth* site; *β_0_* = mean of the cohort height of dominant/codominants when all additional *β’*s = 0; *SD_ij_* = stand density of the *ith* stand within the *jth* site; *DMR*_ij_ = dwarf mistletoe rating of the *ith* stand within the *jth* site; *PROD.L_ij_* = indicator which = 1 when the productivity of the *ith* stand within the *jth* site is low and 0 otherwise; *PROD.M_ij_* = indicator which = 1 when the productivity of the *ith* stand within the *jth* site is moderate and 0 otherwise; *MPBMORT.L_ij_* = indicator which = 1 when the mortality density of the previous mountain pine beetle epidemic of the *ith* stand within the *jth* site is low and 0 otherwise; *MPBMORT.L_ij_* = indicator which = 1 when the mortality density of the previous mountain pine beetle epidemic of the *ith* stand within the *jth* site is moderate and 0 otherwise; *b_j_* = random error for the *jth* site; *b_j_* ~ N(0, σ_b_^2^) and *b_j_* and *b_j’_* are independent; ***ε_ij_*** = random error from the cohort height of dominant/codominants measurements *ith* stand replicate within the *jth* site, ***ε_ij_*** ~ N(0, σ^2^) and ***ε_ij_*** and ***ε_i’j’_*** are independent.
