# Supplementary material for: Effects of Dwarf Mistletoe on Stand Structure of Lodgepole Pine Forests 21-28 Years Post-Mountain Pine Beetle Epidemic in Central Oregon
Source: PLoS One. 2014 Sep 15;9(9):e107532. doi: 10.1371/journal.pone.0107532 (PMC4164639; doi:10.1371/journal.pone.0107532)
Supplement: Table S13 — BIC table for the natural logarithm of cohort diameter of dominant/codominants model. (DOCX) [file pone.0107532.s013.docx]

**Table S13.** BIC table for the natural logarithm of cohort diameter of dominant/codominants model.

| **Model** | **df** | **BIC** | **ΔBIC** | **BIC weight** | **Evidence ratio** |
| --- | --- | --- | --- | --- | --- |
| ***Log(CDD)_ij_ = β_0_ + b_j_ + β_1_DMR_ij_ + β_2_SD_ij_ + ε_ij_*** | 5 | -39.51 | 0 | 5.22E-03 | 1 |
| ***Log(CDD)_ij_ = β_0_ + b_j_ + β_1_SD_ij_ + ε_ij_*** | 4 | -38.24 | 1.27 | 2.77E-03 | 1.89 |
| ***Log(CDD)_ij_ = β_0_ + b_j_ + β_1_DMR_ij_ + β_2_SD_ij_ + β_3_DMR*SD_ij_ + ε_ij_*** | 6 | -37.23 | 2.27 | 1.67E-03 | 3.12 |
| ***Log(CDD)_ij_ = β_0_ + b_j_ + β_1_DMR_ij_ + β_2_SD_ij_ + β_3_MPBMORT.L_ij_ + β_4_MPBMORT.M_ij_ + ε_ij_*** | 7 | -35.08 | 4.43 | 5.70E-04 | 9.16 |
| ***Log(CDD)_ij_ = β_0_ + b_j_ + β_1_DMR_ij_ + β_2_SD_ij_ + β_3_MPBMORT.L_ij_ + β_4_MPBMORT.M_ij_ + β_5_SD*DMR_ij_ + β_6_MPBMORT.L*DMR_ij_ + β_7_MPBMORT.M*DMR_ij_ + ε_ij_*** | 10 | -33.69 | 5.82 | 2.85E-04 | 18.32 |
| ***Log(CDD)_ij_ = β_0_ + b_j_ + β_1_DMR_ij_ + β_2_SD_ij_ + β_3_PROD.L_ij_ + β_4_PROD.M_ij_ + ε_ij_*** | 7 | -32.77 | 6.74 | 1.80E-04 | 29.01 |
| ***Log(CDD)_ij_ = β_0_ + b_j_ + β_1_DMR_ij_ + ε_i_*** | 4 | -31.95 | 7.55 | 1.19E-04 | 43.69 |
| ***Log(CDD)_ij_ = β_0_ + b_j_ + β_1_DMR_ij_ + β_2_MPBMORT.L_ij_ + β_3_MPBMORT.M_ij_ + ε_ij_*** | 6 | -29.41 | 10.10 | 3.34E-05 | 156.14 |
| ***Log(CDD)_ij_ = β_0_ + b_j_ + β_1_DMR_ij_ + β_2_MPBMORT.L_ij_ + β_3_MPBMORT.M_ij_ + β_4_DMR*MPBMORT.L_ij_ + β_5_DMR*MPBMORT.M_ij_ + ε_ij_*** | 8 | -28.40 | 11.10 | 2.03E-05 | 257.63 |
| ***Log(CDD)_ij_ = β_0_ + b_j_ + β_1_DMR_ij_ + β_2_MPBMORT.L_ij_ + β_3_MPBMORT.M_ij_ + β_4_PROD.L_ij_ + β_5_PROD.M_ij_ + β_6_SD_ij_ + ε_ij_*** | 9 | -27.95 | 11.55 | 1.62E-05 | 322.59 |
| ***Log(CDD)_ij_ = β_0_ + b_j_ + β_1_DMR_ij_ + β_2_SD_ij_ + β_3_PROD.L_ij_ + β_4_PROD.M_ij_ + β_5_SD*DMR_ij_ + β_6_PROD.L*DMR_ij_ + β_7_PROD.M*DMR_ij_ + ε_ij_*** | 10 | -27.49 | 12.01 | 1.28E-05 | 406.25 |
| ***Log(CDD)_ij_ = β_0_ + b_j_ + β_1_DMR_ij_ + β_2_PROD.L_ij_ + β_3_PROD.M_ij_ + ε_ij_*** | 6 | -26.59 | 12.92 | 8.17E-06 | 639.11 |
| ***Log(CDD)_ij_ = β_0_ + b_j_ + β_1_DMR_ij_ + β_2_MPBMORT.L_ij_ + β_3_MPBMORT.M_ij_ + β_4_PROD.L_ij_ + β_5_PROD.M_ij_ + β_6_SD_ij_ + β_7_PROD.L*DMR_ij_ + β_8_PROD.M*DMR_ij_ + β_9_MPBMORT.L*DMR_ij_ + β_10_MPBMORT.M*DMR_ij_ + β_11_SD*DMR_ij_ + ε_ij_*** | 14 | -24.26 | 15.25 | 2.55E-06 | 2046.18 |
| ***Log(CDD)_ij_ = β_0_ + b_j_ + β_1_MPBMORT.L_ij_ + β_2_MPBMORT.M_ij_ + ε_ij_*** | 5 | -23.48 | 16.03 | 1.73E-06 | 3021.16 |
| ***Log(CDD)_ij_ = β_0_ + b_j_ + β_1_DMR_ij_ + β_2_PROD.L_ij_ + β_3_PROD.M_ij_ + β_4_DMR*PROD.L_ij_ + β_5_DMR*PROD.M_ij_ + ε_ij_*** | 8 | -22.81 | 16.70 | 1.24E-06 | 4224.97 |
| ***Log(CDD)_ij_ = β_0_ + b_j_ + β_1_PROD.L_ij_ + β_2_PROD.M_ij_ + ε_ij_*** | 5 | -22.70 | 16.81 | 1.17E-06 | 4468.87 |
| ***Log(CDD)_ij_ = β_0_ + b_j_ + β_1_DMR_ij_ + β_2_MPBMORT.L_ij_ + β_3_MPBMORT.M_ij_ + β_4_PROD.L_ij_ + β_5_PROD.L_ij_ + ε_ij_*** | 8 | -22.20 | 17.31 | 9.11E-07 | 5729.23 |
| ***Log(CDD)_ij_ = β_0_ + b_j_ + β_1_DMR_ij_ + β_2_MPBMORT.L_ij_ + β_3_MPBMORT.M_ij_ + β_4_PROD.L_ij_ + β_5_PROD.M_ij_ + β_6_PROD.L*DMR_ij_ + β_7_PROD.M*DMR_ij_ +β_8_MPBMORT.L*DMR_ij_ + β_9_MPBMORT.M*DMR_ij_ + ε_ij_*** | 12 | -15.80 | 23.71 | 3.71E-08 | 140723.61 |

Note: df= degrees of freedom; BIC = Bayesian Information Criterion; ΔBIC = difference in BIC value as compared with that of the preferred model; *Log(CDD)_ij_* = natural logarithm of cohort diameter of dominant/codominants of the *ith* stand within the *jth* site; *β_0_* = mean of the natural logarithm of cohort diameter of dominant/codominants when all additional *β’*s = 0; *SD_ij_* = stand density of the *ith* stand within the *jth* site; *DMR*_ij_ = dwarf mistletoe rating of the *ith* stand within the *jth* site; *PROD.L_ij_* = indicator which = 1 when the productivity of the *ith* stand within the *jth* site is low and 0 otherwise; *PROD.M_ij_* = indicator which = 1 when the productivity of the *ith* stand within the *jth* site is moderate and 0 otherwise; *MPBMORT.L_ij_* = indicator which = 1 when the mortality density of the previous mountain pine beetle epidemic of the *ith* stand within the *jth* site is low and 0 otherwise; *MPBMORT.L_ij_* = indicator which = 1 when the mortality density of the previous mountain pine beetle epidemic of the *ith* stand within the *jth* site is moderate and 0 otherwise; *b_j_* = random error for the *jth* site; *b_j_* ~ N(0, σ_b_^2^) and *b_j_* and *b_j’_* are independent; ***ε_ij_*** = random error from the natural logarithm of cohort diameter of dominant/codominants measurements *ith* stand replicate within the *jth* site, ***ε_ij_*** ~ N(0, σ^2^) and ***ε_ij_*** and ***ε_i’j’_*** are independent.
